# Epstein-Barr virus infection induces tissue-resident memory T cells in mucosal lymphoid tissues

**DOI:** 10.1172/jci.insight.173489

**Published:** 2024-10-22

**Authors:** Daniel Kirchmeier, Yun Deng, Lisa Rieble, Michelle Böni, Fabienne Läderach, Patrick Schuhmachers, Alma Delia Valencia-Camargo, Anita Murer, Nicole Caduff, Bithi Chatterjee, Obinna Chijioke, Kyra Zens, Christian Münz

**Affiliations:** 1 Viral Immunobiology, Institute of Experimental Immunology, and; 2 Cellular Immunotherapy, Institute of Experimental Immunology, University of Zürich, Zurich, Switzerland.; 3 Institute of Medical Genetics and Pathology, University Hospital Basel, Basel, Switzerland.

**Keywords:** Immunology, Infectious disease, Adaptive immunity, Cellular immune response, T cells

## Abstract

EBV contributes to around 2% of all tumors worldwide. Simultaneously, more than 90% of healthy human adults persistently carry EBV without clinical symptoms. In most EBV carriers, it is thought that virus-induced tumorigenesis is prevented by cell-mediated immunity. Specifically, memory CD8^+^ T cells recognize EBV-infected cells during latent and lytic infection. Using a symptomatic primary infection model, similar to infectious mononucleosis (IM), we found EBV-induced CD8^+^ tissue resident memory T cells (TRMs) in mice with a humanized immune system. These human TRMs were preferentially established after intranasal EBV infection in nasal-associated lymphoid tissues (NALT), equivalent to tonsils, the primary site of EBV infection in humans. They expressed canonical TRM markers, including CD69, CD103, and BLIMP-1, as well as granzyme B, CD107a, and CCL5. Despite cytotoxic activity and cytokine production ex vivo, these TRMs demonstrated reduced CD27 expression and proliferation and failed to control EBV viral loads in the NALT during infection, although effector memory T cells (TEMs) controlled viral titers in spleen and blood. Overall, TRMs are established in mucosal lymphoid tissues by EBV infection, but primarily, systemic CD8^+^ T cell expansion seems to control viral loads in the context of IM-like infection.

## Abstract 2

Epstein Barr virus (EBV) infection induces tissue-resident cytotoxic CD8+ T cells in mucosal secondary lymphoid tissues, that are, however, not able to control viral loads.

## Graphical abstract

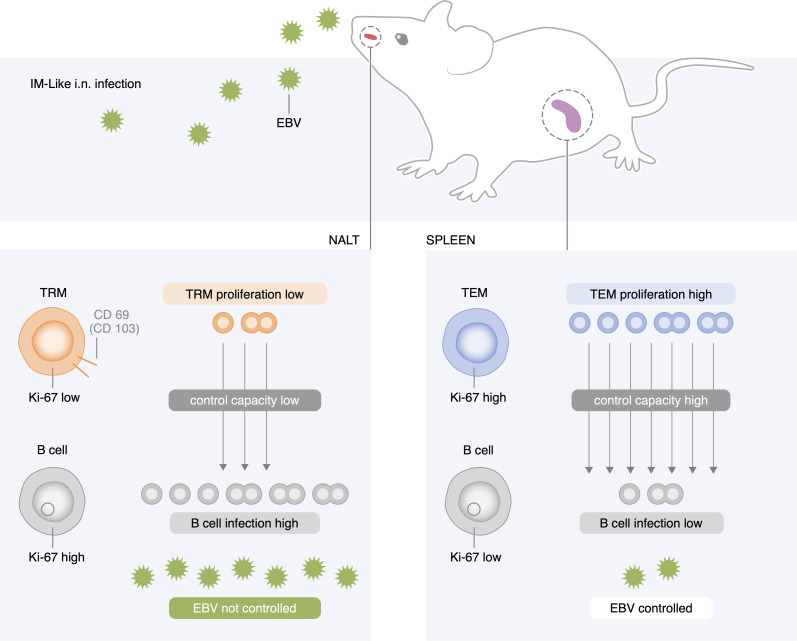

## Introduction

EBV, or human herpesvirus 4 (HHV4), is a WHO class I carcinogen and associated with approximately 2% of all tumors in humans (1). Although more than 90% of the human adult population is persistently infected with EBV, fortunately only a small number of virus carriers are affected by the approximately 300,000 new EBV-associated malignancies that arise every year (2–4). EBV is usually transmitted via saliva exchange and infects its main host, the human B cell, in mucosal secondary lymphoid tissues, such as the tonsils, presumably after transcytosis across mucosal epithelia (5, 6). Initially, EBV expresses only latent gene products in B cells that are thought to drive host cell differentiation into the memory B cell pool (7). These EBV nuclear antigens (EBNAs) and latent membrane proteins (LMPs) are then switched off in quiescent memory B cells, which then function as the cellular compartment of long-term viral persistence. Successive antigen-driven B cell differentiation into plasma cells induces lytic EBV replication (8, 9). Although most primary EBV infections are asymptomatic, delayed first encounter with the virus in adolescence or young adulthood increases the risk of developing infectious mononucleosis (IM), a massive CD8^+^ T cell lymphocytosis that is mainly driven by lytic EBV antigens (10, 11). IM usually peaks 6 weeks after virus encounter (12). Although IM resolves to a persistent, immune-controlled EBV infection in most affected individuals, it nevertheless increases the risk of developing both virus-associated classic Hodgkin’s lymphoma, one of the EBV-associated malignancies, and the autoimmune disease multiple sclerosis (MS) (13, 14). It remains unclear why delayed primary EBV infection increases the risk for IM. High infectious dose or poor and delayed immune control at the initial site of virus encounter, namely mucosal secondary lymphoid tissues such as tonsils, might allow high lytic viral replication. Subsequently this could trigger the systemic CD8^+^ T cell lymphocytosis that causes IM. In order to develop potential therapeutic strategies to alleviate IM, it is important to better understand EBV-specific immune responses during initial infection at mucosal surfaces.

Tissue-resident memory T cells (TRMs) comprise a distinct subset of memory T cells residing stably within tissues. They are best described in the context of pathogens causing self-limiting, acute illnesses and are thought to play central roles in protection from reinfection (15–17). To this extent, a substantial fraction of memory T cells in mucosal tissues are tissue resident and located at major sites of pathogen encounter. Mucosal TRMs are typically distinguished by expression of CD69 and often coexpress the epithelial adhesion molecule CD103 (18–21). While TRMs are generally considered to be important mediators of protective immunity, aberrant TRM generation has been associated with certain pathologies, including asthma, psoriasis, vitiligo, Crohn’s disease, and ulcerative colitis (22–26). Furthermore, reduced TRM establishment and function are associated with poor patient outcomes in certain solid cancers, including lung and breast cancers (27–30), and may contribute to reduced control of chronic infections such as human immunodeficiency virus (HIV), hepatitis B virus (HBV), and hepatitis C virus (HCV) (31–34). TRMs are often thought to represent a terminal state of memory differentiation after central memory T cells (TCMs) and effector memory T cells (TEMs). Inflammatory conditions skew T cell differentiation toward TEM, whereas less inflammatory conditions tend to favor TCM primed for circulation and long-term survival (35). In chronic and persisting infections specifically, it is unclear whether high levels of inflammation or continuous and repeated antigen exposures may negatively affect TRM generation or otherwise reduce their functional capacities.

EBV-specific T cells with a TRM phenotype have been previously described in humans (36–38), notably in the tonsils, which may represent a frontline site in the control of viral reactivation and prevention of viral shedding. However, examinations of donor tissues only represent a snapshot in the infection process, and the generation and function of EBV-specific memory T cells remain incompletely characterized. To this extent, humanized mice, which are engrafted with human lymphocytes and are permissive to EBV infection, represent an ideal model to study T cell response dynamics. Since antigen location is further known to be important in the establishment of certain TRM subsets, we have developed an intranasal model of EBV infection to better recapitulate the natural route of viral exposure. Using this model, we have observed the generation of TRMs following EBV infection preferentially in nasal-associated lymphoid tissue (NALT), the murine tonsil equivalent (39). Despite phenotypic similarities to previously described TRM populations and similar functionality in vitro, depletion of CD8^+^ T cells during EBV infection resulted in increased blood and splenic viral loads, while depletion of CD103^+^ TRMs did not influence EBV infection in the NALT. Our data suggest that differences in proliferative capacity and costimulation render TRMs less protective during EBV-associated IM, a persisting and comparatively inflammatory infection.

## Results

### EBV initiates infection in NALT after i.n. inoculation.

Here we used human immune system reconstituted (humanized) NOD/SCID/IL-2R^null^ (NSG) mice and NSG mice with an HLA-A*02:01 transgene (NSG-A2) to investigate cell-mediated immune control of EBV infection (40–42). Using these models, our group previously identified protective roles for circulating CD27^+^2B4^+^CD8^+^ T cells in EBV control (43–46). The establishment and role of mucosal CD8^+^ T cells during EBV infection have, however, not been assessed. To evaluate mucosal T cell responses, we modeled salivary EBV infection by i.n. inoculation with the B95-8 strain expressing luciferase to aid in visualization of infection (46, 47), initially alongside the standard i.p. route of infection as a control. To ensure consistent NALT establishment and infection, we mimicked the bacterial colonization driving postnatal NALT development (48, 49) by pretreating animals with a single i.n. dose of Staphylococcus enterotoxin B (SEB) 2 weeks prior to infection (Supplemental Figure 1A; supplemental material available online with this article; https://doi.org/10.1172/jci.insight.173489DS1). IHC analysis of resulting NALTs (Supplemental Figure 1B) revealed expression of Lyve-1 and peripheral node addressin (PNAd), indicative of lymphatic vessel and high endothelial venule-like structures (Supplemental Figure 1D), as well as the accumulation of CD3^+^ and CD20^+^ lymphocytes with a similar T/B cell ratio as human tonsils (Supplemental Figure 1, B and C).

**Figure 1:**
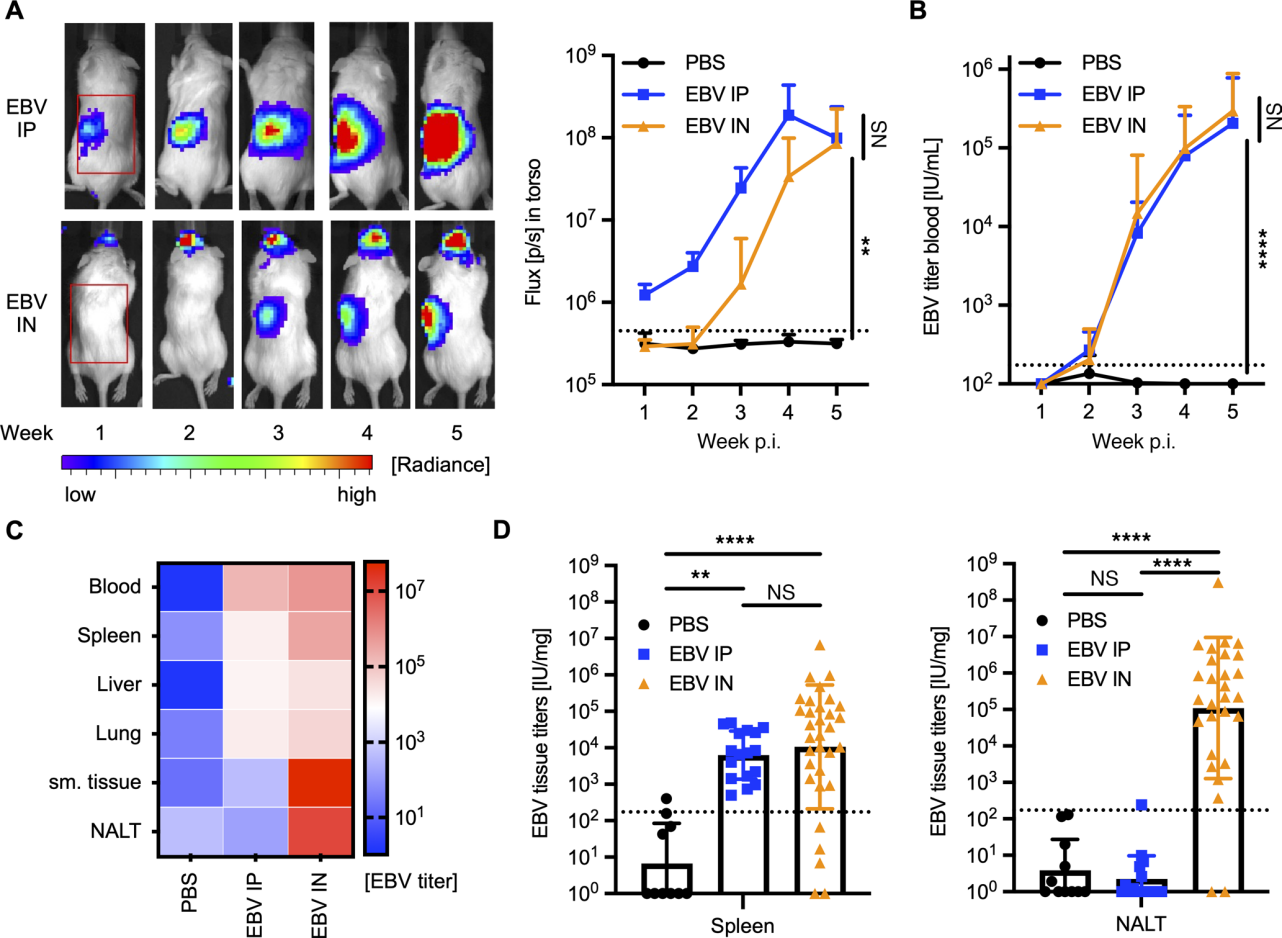
EBV is present in submandibular tissue and NALT following intranasal infection. (**A**) Representative IVIS image analysis over 5 weeks of Luc-EBV infection (left) with (right) quantification of the outlined region of interest (*n* = 6–24 animals per group from 3–6 independent experiments). EBV IP, i.p. infection; EBV IN, i.n. infection. (**B**) EBV blood viral loads over time in IU/mL in infected animals or PBS controls (*n* = 4–31 animals per group from 3–6 independent experiments; quantification in Supplemental Figure 2B). (**C**) Heatmap plot for EBV viral loads in blood and indicated tissues at sacrifice (*n* = 3–32 animals per group from 3–6 independent experiments; quantification in Supplemental Figure 2C). (**D**) Quantification of viral loads in spleen (left) and NALT (right) (*n* = 9–33 animals per group from 3-6 independent experiments). **P ≤ 0.01, ****P ≤ 0.0001, by Kruskal-Wallis test with Dunn’s test for multiple comparisons; dotted line indicates limit of detection.

Following i.n. EBV infection, luciferase signal was detectable within the first week after infection and localized initially to the NALT (Figure 1A and Supplemental Figure 2A). From there, we observed viral spread to the submandibular tissues, including submandibular and cervical lymph nodes (draining the palatine of the nasopharyngeal cavity, head, and neck) and salivary glands (Figure 1A). Systemic dissemination into the torso was visible from week 3 onward (Figure 1A and Supplemental Figure 2A). In contrast, luciferase signal in the NALT region was rarely detected following i.p. infection (Supplemental Figure 2A). We further assessed viral loads in the blood and tissues. Viral loads in the blood had similar kinetics during both i.n. and i.p. infection (Figure 1B). In the tissues, EBV was detected in all highly blood-perfused organs, including spleen, liver, and lung, following both infection routes (Figure 1, C and D, and Supplemental Figure 2B) but only in submandibular tissues (including submandibular and cervical lymph nodes) and NALT following i.n. infection (Figure 1, C and D, and Supplemental Figure 2B). Similar patterns of infection were observed in both humanized NSG and NSG-A2 mice (Supplemental Figure 2C). Thus, following i.n. infection, EBV first colonized secondary lymphoid tissues of the nasopharyngeal mucosa before systemically disseminating to the blood and spleen, consistent with the physiologic route of human infection.

### EBV induces both systemic T cell responses and NALT TRMs upon i.n. infection.

**Figure 2:**
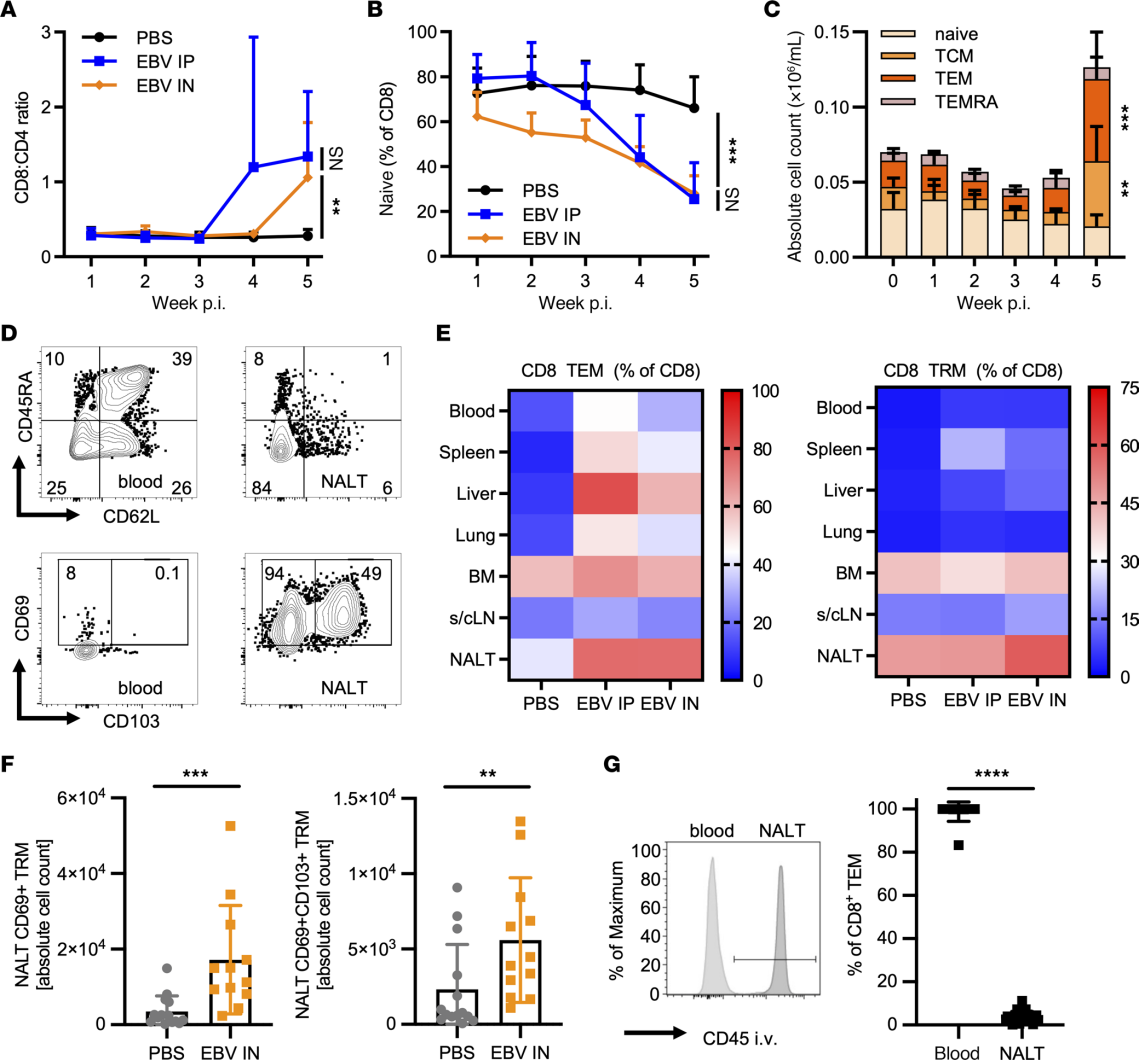
I.n. EBV infection induces TRMs in the NALT. (**A** and **B**) Ratio of CD8/CD4 T cell frequencies and frequency of naive (CD45RA^+^CD62L^+^) CD8^+^ T cells in blood over time. (**C**) Numbers of naive, central memory (TCM; CD45RA^–^CD62L^+^), effector memory (TEM; CD45RA^–^CD62L^–^), and TEMRA (CD45RA^+^CD62L^–^) CD8^+^ T cells in blood over time (*n* = 2–9 animals per group depicted as mean values with 95% CI). (**D**) Representative flow cytometry plots and gating for CD45RA and CD62L of CD8^+^ T cells and (top row) and (bottom row) for CD69 and CD103 of CD8^+^ TEM T cells in blood and NALT at sacrifice. (**E**) Heatmap of frequencies of CD8^+^ TEM (left) and TRM (right) (CD69^+^ TEM) T cells in indicated tissues at sacrifice (*n* = 4–34 animals per group from a minimum of 2 independent experiments; quantification in Supplemental Figures 3, A and B). (**F**) Quantification of absolute cell numbers of CD69^+^ TEM (left) and CD69^+^CD103^+^ TEM (right) in the NALT in PBS versus i.n. infected mice. (**G**) Representative histogram of geometric mean fluorescence intensity (MFI) of i.v. injected anti-CD45 in blood and NALT (left) and quantification (right) (*n* = 14–28 animals per group from a minimum of 2 independent experiments). **P ≤ 0.01, ***P ≤ 0.001, ****P ≤ 0.0001, by Kruskal-Wallis test for 3 groups (**A** and **B**) or Mann Whitney *U* test for comparison of 2 groups (**F** and **G**), or using paired Wilcoxon signed-rank test comparing T cell subsets between weeks 4 and 5 (**C**).

Route of infection can influence T cell differentiation and subsequent protective capacities (50). Therefore, we were interested in comparing T cell phenotypes following i.n. or i.p. infection. For both infection routes, the CD8^+^/CD4^+^ T cell ratio in peripheral blood increased progressively over 5 weeks of infection, indicating CD8^+^ T cell–dominated responses (Figure 2A). Thus, we focused our analyses on CD8^+^ T cells. Following both i.n. and i.p. infection, CD8^+^ T cells shifted away from a CD45RA^+^CD62L^+^ naive phenotype (Figure 2, B and C), with concomitant expansion of CD45RA^–^CD62L^+^ TCMs and CD45RA^–^CD62L^–^ TEMs (Figure 2C). For both infection routes, we further observed high frequencies of TEMs in highly blood perfused tissues, including spleen, liver, and lung, as well as in mucosal tissues, including the NALT, at sacrifice (Figure 2, D and E, and Supplemental Figure 3A), indicating that activated effectors traffic throughout much of the body during infection. Interestingly, following i.n. EBV infection, both frequencies and numbers of CD69^+^ and CD69^+^CD103^+^ CD8^+^ T cells were increased in the NALT at sacrifice (Figure 2, D–F, and Supplemental Figure 3A). Some CD8^+^ and CD103^+^ cells were found in close proximity to cells expressing EBV-encoded small RNAs (EBERs) in the NALT (Supplemental Figure 1, F and G). These cells were predominantly intravascular antibody negative (Figure 2G), indicating localization to the extravascular space, typical of TRMs (51).

In addition to evaluating polyclonal responses, we utilized an adoptive transfer model to confirm the establishment of EBV-specific CD8^+^ TRMs following infection. We transduced spleen-derived T cells with HLA-A*02:01 restricted EBV-specific T cell receptors (TCRs) recognizing either lytic BMLF1 or latent LMP2 antigens, as described previously (45, 46). These EBV-specific TCR transgenic T cells were then adoptively transferred into autologously reconstituted littermate animals. Transduced cells were identified by expression of both the respective HLA-A*02:01/peptide pentamer and the murine membrane-proximal TCR domain (due to the hybrid format of the transgenic TCRs; Supplemental Figure 3B). TCR transgenic cells migrated to the NALT were intravascular antibody negative (Supplemental Figure 3C) and generated both CD69^+^ and CD69^+^CD103^+^ populations, similar to the overall effector response (Supplemental Figure 3D). Thus, while both i.p. and i.n. EBV infection elicit systemic TEM responses, i.n. infection selectively expands T cells with a TRM phenotype in the NALT.

### Single-cell RNA-Seq of NALT T cells reveals TRM transcriptome.

**Figure 3:**
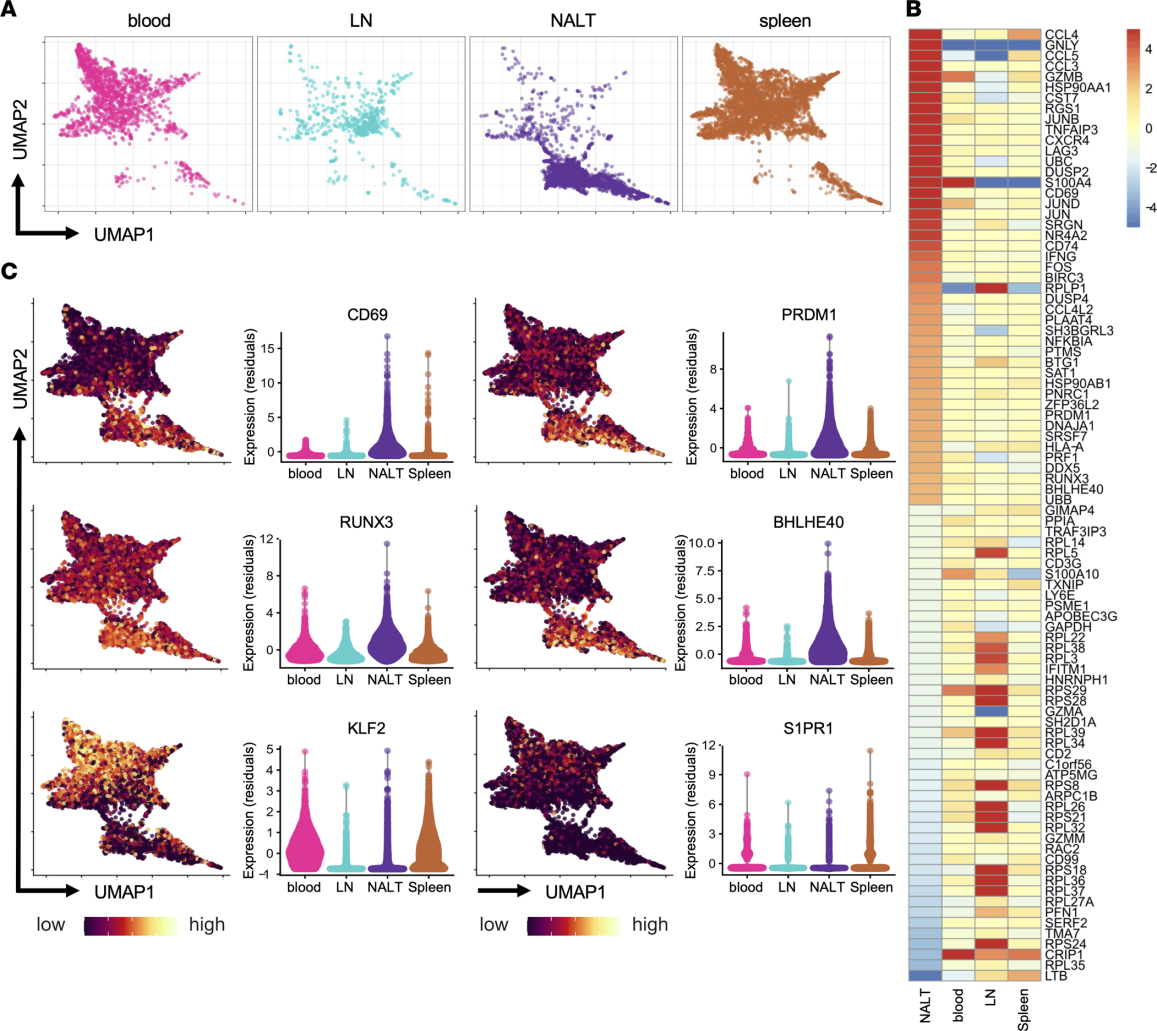
scRNA-Seq reveals TRM-like transcriptome of NALT CD8^+^ T cells following i.n. EBV infection. (**A**) UMAP plot of single CD8^+^ TEM transcriptomes sorted from indicated tissues. (**B**) Heatmap showing log_2_(fold change) of the top 50 highest and lowest differentially expressed genes of NALT single-cell transcriptomes (rows). (**C**) Integrated UMAP plots of cells showing relative expression of indicated marker expression (first and third columns; yellow-green, high expression; violet, low expression) and (second and fourth columns) violin plot depicting single-cell expression of markers in indicated tissues as normalized residual values.

To characterize the transcriptional profile of the NALT T cells elicited by i.n. infection, we performed single-cell RNA-Seq (scRNA-Seq) of CD8^+^ TEMs sorted from blood, spleen, submandibular, and cervical LNs and NALT of EBV-infected animals after 6.5 weeks of infection. This allowed us to compare transcriptional patterns between responding T cells from the NALT and T cells from organs not harboring a pronounced TRM phenotype during infection. Uniform Manifold Approximation and Projection (UMAP) plots of the scRNA-Seq data reveal that NALT cells were transcriptionally distinct from those of the other 3 tissues (Figure 3A). By differential expression analyses (52–54), we identified the 50 genes most up- and downregulated by NALT cells, compared with those of other tissues (Figure 3B). Genes previously reported to be important for TRM development, including *RUNX3*, *BHLHE40*, and *PRDM1* (encoding BLIMP-1), as well as TRM core signature genes, including *PDCD1* (encoding PD-1) and *CD69*, were upregulated in NALT-derived T cells (55–58). Genes encoding chemokines, cytokines, and cytotoxic molecules, including *CCL3*, *CCL4*, *CCL5*, *IFNG*, and *GZMB*, were also elevated in NALT-derived cells, as were *CXCR4* and *LAG3* (Figure 3B). When plotting selected markers of interest onto the UMAP, TRM-related gene expression was predominately increased in NALT cells, while *KLF2* and *S1PR1*, associated with tissue egress, were more highly expressed in cells derived from the blood and spleen (Figure 3C) (59). Altogether, these data support that NALT-derived T cells responding to EBV infection have a TRM-like transcriptional profile and likely represent a bona fide population of TRMs that is generated in this tissue in response to EBV infection.

### Ontogeny of NALT TRMs.

**Figure 4:**
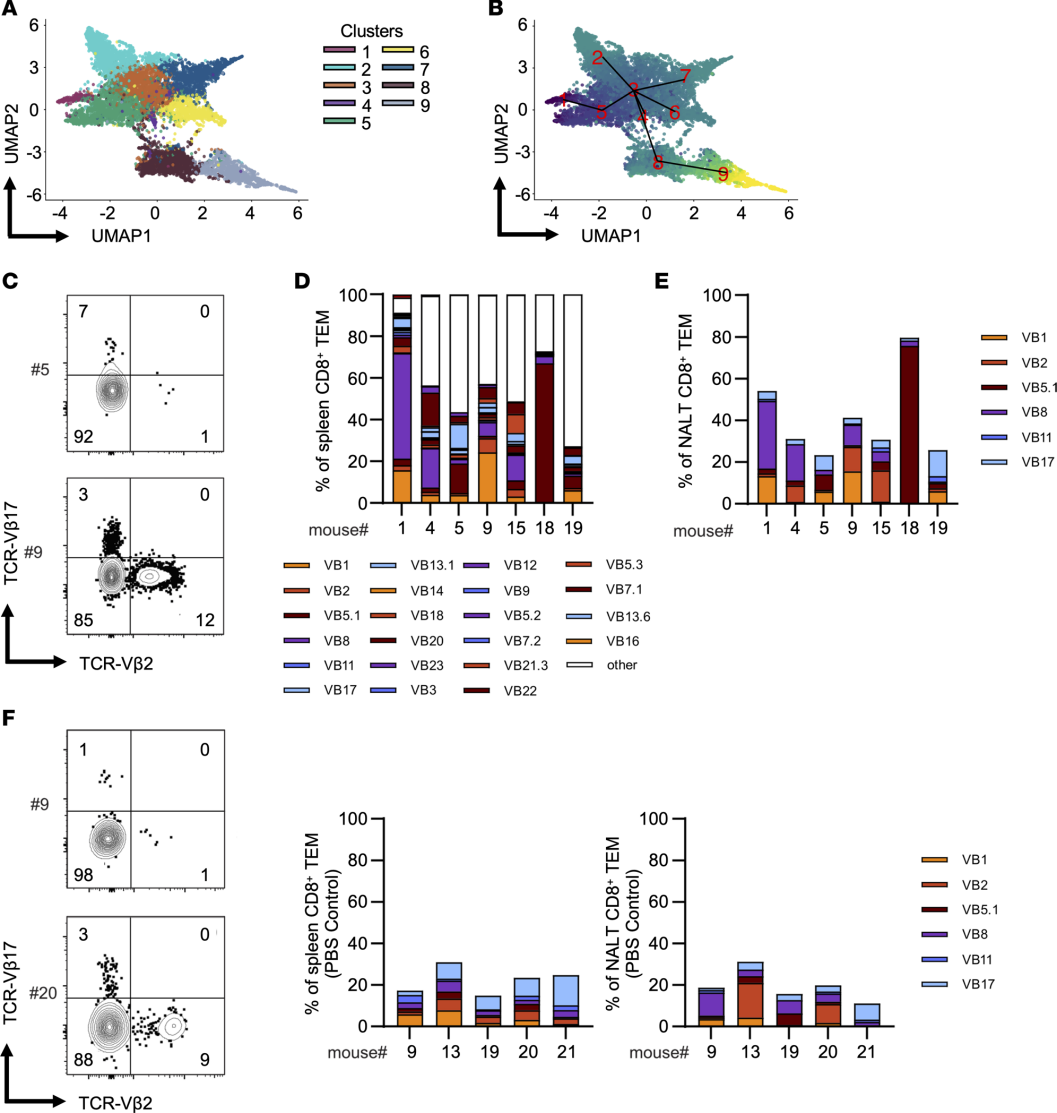
Ontology of NALT TRM following i.n. EBV infection. (**A**) Integrated UMAP plot depicting unsupervised clustering of single CD8^+^ TEM transcriptomes sorted from blood, LN, NALT, and spleen. (**B**) Cellular trajectories in pseudo time (blue to yellow, earlier to later pseudo time point) of single CD8^+^ TEM transcriptomes sorted from blood, LN, NALT, and spleen. (**C**) Representative flow cytometry plots of example TCR-Vβ17 and TCR-Vβ2 staining in 2 EBV-infected animals. (**D**) Frequencies of indicated TCR-VB clones among splenic CD8^+^ TEMs following intranasal EBV infection. (**E**) Frequencies of the top 6 TCR-VB clones in NALT CD8^+^ TEMs in the same animals. (**F**) Representative flow cytometry plots of example TCR-Vβ17 and TCR-Vβ2 staining in 2 PBS control animals (left) frequencies of the top 6 TCR-VB clones in spleen CD8^+^ TEMs of PBS control animals (center) and frequencies of the top 6 TCR-VB clones in NALT CD8^+^ TEMs of PBS control animals (right).

From transcriptional data, using an unsupervised clustering algorithm (52), we identified 9 distinct clusters within the 4 tissues, including 2 clusters within the NALT (Figure 4A and Supplemental Figure 4A), though patterns of differential gene expression were comparable within these 2 clusters (Supplemental Figure 4B). We computed pseudotemporal cell relationships between the identified clusters using the Tools for Single-Cell Analysis (TSCAN) algorithm to evaluate cellular developmental trajectories (53). We found that the 2 NALT clusters were likely derived from clusters in the center of the UMAP (Figure 4B), suggesting that they differentiated from other cell populations shortly after migrating into the NALT. To further investigate the relationship between T cells from NALT and spleen, we analyzed TCR-Vβ repertoires from infected animals (Figure 4C). Interestingly, we found that clonal CD8^+^ T cell expansion in the spleen differed substantially between individual EBV-infected mice (Figure 4D). Using the 6 most commonly detected TCR-Vβ chains in the spleen, we analyzed the overlap in the NALT of the same animals. We observed that the most expanded TCR-Vβ chains within individual mice overlapped in both spleen and NALT (Figure 4E). Furthermore, these clones differed substantially from those in the spleen or NALT of PBS control mice, indicating EBV-specific T cell expansion (Figure 4F). Combined with our pseudotemporal gene expression analysis, these data are consistent with a model where, upon EBV infection, a given virus-specific T cell is clonally expanded and gives rise to daughters that then populate both the NALT, where they differentiate into TRM, and the spleen, where they remain predominantly TEM.

### Comparison of TRM markers between NALT of EBV-infected humanized mice and human tonsils.

**Figure 5:**
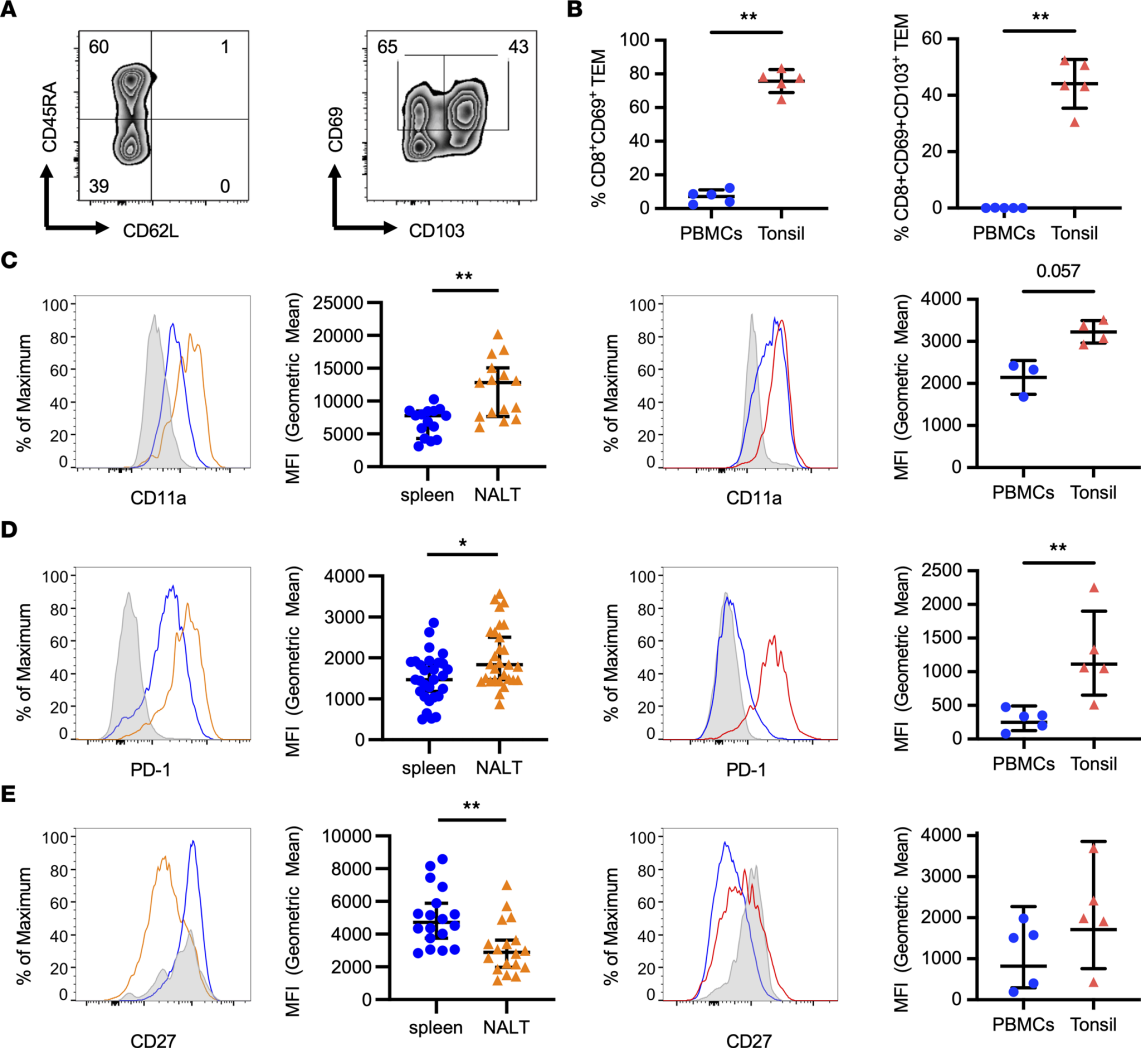
Similarities in TRM marker expression between CD8^+^ T cells from EBV-infected NALT and tonsils from EBV^+^ donors. (**A**) Representative flow cytometry gating for CD8^+^ TEM and CD69^+^ and CD69^+^CD103^+^ CD8^+^ TEM in human tonsils. (**B**) Quantification of CD8^+^ TEM and CD69^+^ and CD69^+^CD103^+^ CD8^+^ TEM in human PBMCs and tonsils. (**C**) Representative histogram of geometric mean fluorescence intensity (MFI) and quantification of CD11a expression by TEM from spleen (blue) and NALT (orange) of EBV-infected animals (left 2 panels) and (right 2 panels) in TEM from human PBMCs (blue) and tonsils from EBV^+^ donors (red). (**D**) MFI and quantification of PD1 expression by TEM in spleen (blue) and NALT (orange) of EBV-infected animals (left 2 panels) and (right 2 panels) in TEM from human PBMCs (blue) and tonsils from EBV^+^ donors (red). (**E**) MFI and quantification of CD27 expression by TEM in spleen (blue) and NALT (orange) of EBV-infected animals (left 2 panels) and (right 2 panels) in TEM from human PBMCs (blue) and tonsils from EBV-positive donors (red). For animal data, *n* = 14–30 animals per group from at least 6 independent experiments; for human data, *n* = 3–5 individuals per tissue from 2 independent experiments, except CD11a data, which is derived from a single experiment. *P ≤ 0.05, **P ≤ 0.01, by Wilcoxon matched-pairs signed-rank test for animal data (second column, **C**, **D**, and **E**) and Mann-Whitney *U* test for human data (**B** and fourth column **C**, **D**, and **E**).

We were additionally interested in the similarities of NALT T cells from EBV-infected humanized mice with T cells from tonsils of healthy human EBV carriers that contain a similar T/B cell ratio (Supplemental Figure 1C). Thus, we analyzed NALT and spleen samples alongside tonsil samples collected from EBV^+^ organ donors or tonsillectomies, as well as blood-derived PBMCs, investigating their CD8^+^ T cell and TRM phenotypes. Of CD8^+^ T cells, TEMs reexpressing CD45RA (TEMRAs) and TEMs were the most commonly observed subsets in human tonsils (Figure 5A), with the majority of tonsillar TEMs also expressing CD69 or CD69 and CD103 (Figure 5B), in contrast to PBMCs, which were predominantly negative for both CD69 and CD103, as expected (Figure 5B). Expression of the TRM-related markers CD11a and PD-1 was also increased in tonsils, as well as in NALT, with expression in both of these tissues elevated compared with spleen or peripheral blood (Figure 5, C and D). Intracellular transcription factors and chemokines partially overlapped between tonsils and NALT. In particular, the hallmark TRM transcription factor BLIMP-1 was highly expressed in the NALT but in only a subset of tonsillar cells (Supplemental Figure 5A). Cytotoxic molecules and chemokines, including granzyme B and CCL5, were also more prominently expressed in the NALT, and CCL5 expression was increased in tonsils as well (Supplemental Figure 5, B and C). NALT-derived cells further lacked expression of the tissue egress marker S1PR1 (Supplemental Figure 5D). Interestingly, in the NALT, expression of the costimulatory molecule CD27, required for efficient immune control of EBV (46), was lower than in spleens of EBV-infected animals (Figure 5E). In the tonsils, CD27 expression was intermediate, lower than that observed for naive CD8^+^ T cells but higher than that of CD8^+^ TEMs in the blood (Figure 5E). Consistent with this, CD8^+^ TEMs in the blood of healthy human donors are primarily specific for other pathogens that drive CD27^–^ memory T cell differentiation (60, 61). Therefore, primary EBV infection appears to drive the establishment of chemokine- and cytokine-producing TRMs in the NALT but seems to diminish expression of CD27, known to be important for immune control of EBV.

### EBV-induced NALT TEMs are functional in vitro.

**Figure 6:**
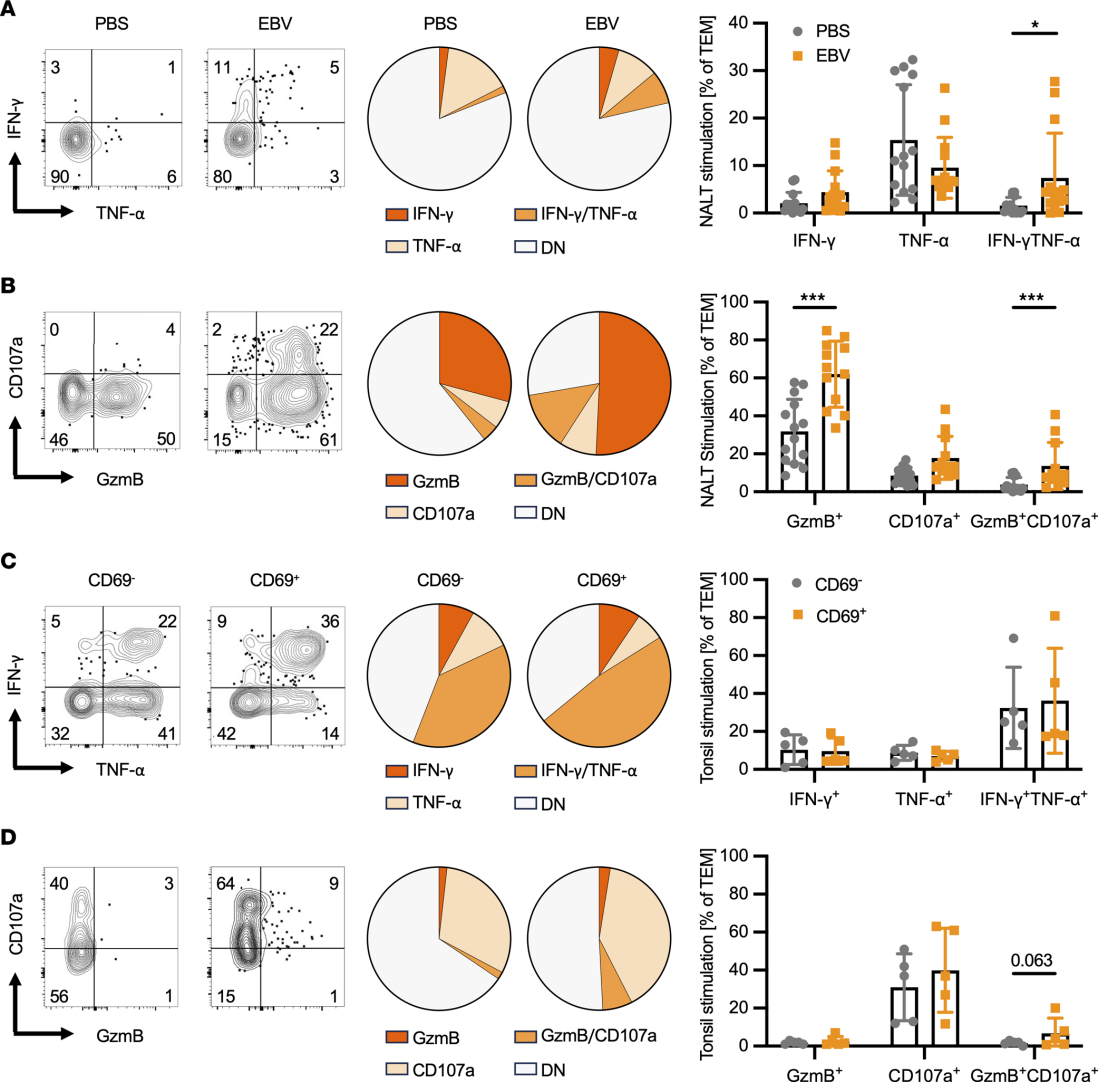
Function of TEM derived from NALT during i.n. EBV infection or from tonsils of EBV carriers. (**A**) Representative flow cytometry plots showing IFN-γ and TNF-α expression by CD8^+^ TEMs derived from PBS control or EBV-infected NALT after PMA/ionomycin stimulation (left panels), as well as the distribution (center panels) and quantification (right panels) of each population. (**B**) As for **A** but depicting CD107a and granzyme B (GzmB) expression. (**C**) Representative flow cytometry plots showing IFN-γ and TNF-α expression byCD69^–^ or CD69^+^ CD8^+^ TEMs derived from tonsils of EBV carriers after PMA/ionomycin stimulation (left panels), as well as the distribution (center panels) and quantification (right panels) of each population. (**D**) As for **C**, but depicting CD107a and GzmB expression. Corresponding unstimulated data for all samples can be found in Supplemental Figure 6. For animal data, *n* = 12–14 animals per group from 2 independent experiments; for human data, *n* = 3–5 individuals per tissue from 2 independent experiments. *P ≤ 0.05, ***P ≤ 0.001, by Wilcoxon matched-pairs signed-rank test for animal data (**A** and **B**) and Mann-Whitney *U* test for human data (**C** and **D**).

We next compared the functional capacities of CD8^+^ T cells derived from the NALT (predominantly CD69^+^ TRMs) and spleen (predominantly CD69^–^ TEMs) of EBV-infected animals or PBS controls, as well as of sorted CD8^+^CD69^–^ and CD8^+^CD69^+^ TEM and TRM, respectively, from tonsils of healthy human EBV carriers. Following ex vivo stimulation, NALT-derived cells from infected animals demonstrated increased IFN-γ and TNF-α expression compared with noninfected PBS controls (Figure 6A and Supplemental Figure 6A). Expression of granzyme B was increased, and a subset of these cells further expressed CD107a, indicating enhanced cytolytic and degranulation capacities (Figure 6B and Supplemental Figure 6B). Similarly, tonsillar TEMs and TRMs coexpressed IFN-γ and TNF-α following stimulation, as well as high levels of CD107a with a trend toward coexpression of CD107a and granzyme B in tonsillar TRMs; this was also observed in NALT-derived cells (Figure 6, C and D, and Supplemental Figure 6, C and D). Together, these data suggest that both NALT and tonsillar TRMs are multifunctional and possess the ability to degranulate and express cytolytic molecules.

### T cell depletion leads to increased EBV viral loads in blood and spleen, but not in the NALT.

**Figure 7:**
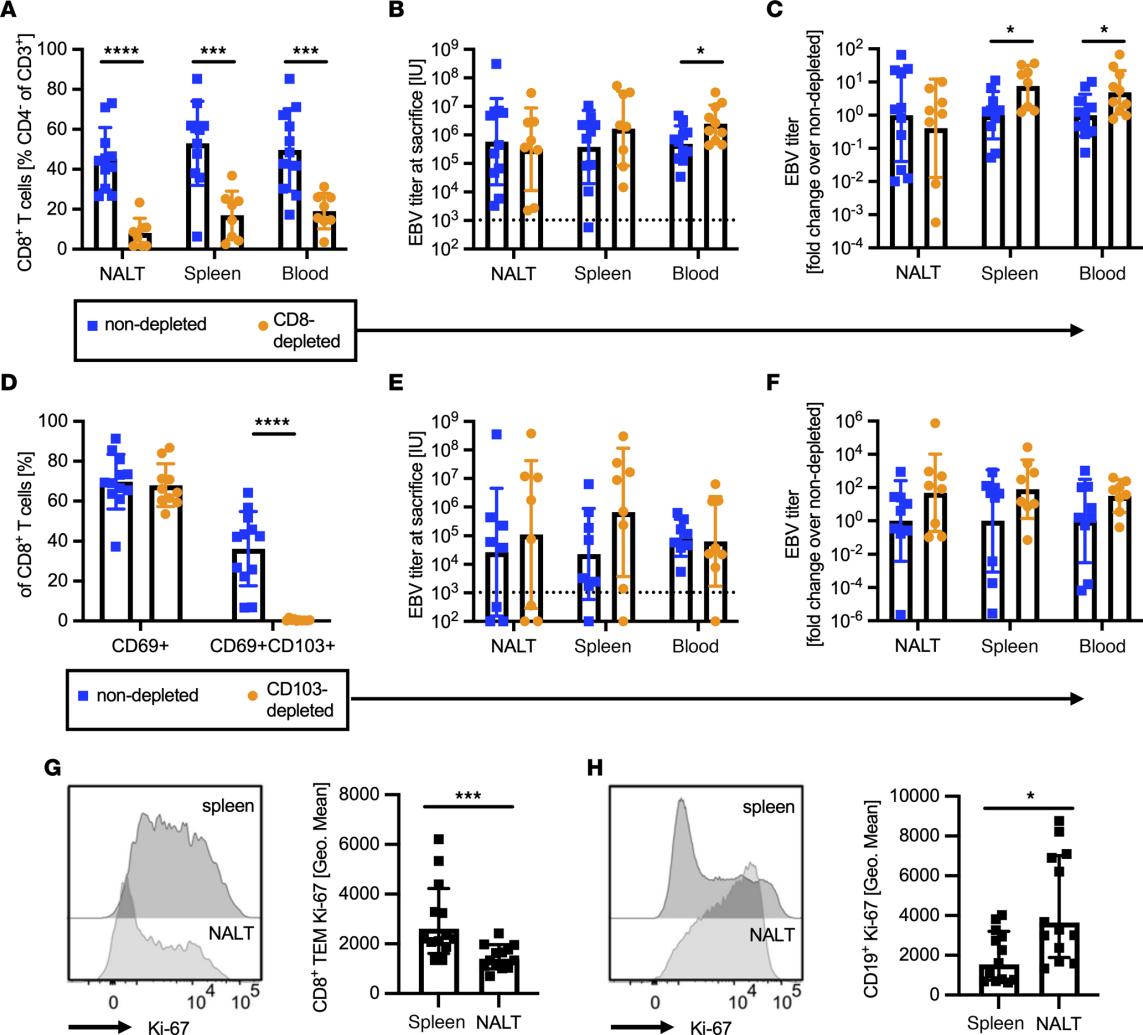
Circulating CD8^+^ T cells, but not NALT TRM, control EBV viral loads. (**A**) CD8^+^ T cell depletion efficiency in NALT, spleen, and blood of control (blue) and OKT8-treated (orange) animals given as percentage of CD4^–^CD3^+^ T cells (*n* = 8–11 animals per group from 2 independent experiments). (**B** and **C**) EBV viral loads in IU/mg NALT, IU/1 × 10^6^ splenocytes, or IU/mL blood and normalized to the mean of the corresponding nondepleted group. (**D**) CD103 depletion efficiency in NALT, spleen, and blood of control (blue) and Ber-OCT3–treated (orange) animals given as percentage of CD8^+^ T cells (*n* = 10–11 animals per group from 2 independent experiments). (**E** and **F**) EBV viral loads in IU/mg NALT, IU/1x × 10^6^ splenocytes, or IU/mL blood and normalized to the mean of the corresponding nondepleted group. (**G**) Representative histogram of Ki-67 expression on CD8^+^ TEM in spleen and NALT (left) and quantification (right). (**H**) Representative histogram of Ki-67 expression on CD19^+^ cells in spleen and NALT (left) and quantification (right) (*n* = 13 animals per group from 2 independent experiments). *P ≤ 0.05, ***P ≤ 0.001, ****P ≤ 0.0001. (**A**–**F**) Mann Whitney *U* test. (**G** and **H**) Wilcoxon matched-pairs signed rank test; dotted line indicates limit of detection.

After characterizing cytokine production and cytolytic potential of NALT TRMs, we next assessed their functional role in vivo by antibody-mediated depletion. To permit the initial accumulation of TRMs in the NALT after infection, we depleted CD8^+^ T cells by OKT-8 antibody injection beginning at week 3 after intranasal EBV administration. In order to avoid any potential bias due to downregulation of CD8 expression, we assessed CD4^–^CD3^+^ T cell frequencies as a measure for CD8^+^ T cell depletion efficacy. CD8^+^ T cells in NALT, spleen, and blood were significantly reduced in treated animals (Figure 7A) indicating that, in contrast to intravascular antibody labeling, which is of a short duration, prolonged treatment with CD8-depleting antibody efficiently targets NALT CD8^+^ T cells for depletion. Strikingly, depletion did not affect viral titers in the NALT (Figure 7, B and C), although viral loads increased markedly in both the spleen and blood of treated animals (Figure 7, B and C). To confirm the limited effect of NALT TRMs on EBV control, we specifically targeted CD103^+^ TRMs, which comprise a major part of the TRMs in the NALT (Figure 2D), by treatment with an anti-CD103 antibody (Ber-ACT8) beginning 3 weeks after i.n. EBV infection. We observed a substantial decrease in CD69^+^CD103^+^ double-positive NALT TRMs, although the frequency of CD69^+^ single-positive TRMs did not change (Figure 7D). Despite this reduction, however, viral loads in the NALT, spleen, and blood did not change, consistent with the results from the systemic T cell depletion approach, with TEM in the spleen and blood controlling EBV at these sites but with NALT TRM having limited immune control (Figure 7, E and F). To decipher further functional differences between lymphocytes within NALT and spleen, we assessed proliferative capacities of T and B cells in both organs. Interestingly, our findings reveal that NALT TRMs exhibit substantially lower expression of the proliferation marker Ki-67 compared with splenic TEMs (Figure 7G). In contrast, B cells in the NALT demonstrate significantly higher virus driven Ki-67 expression compared with B cells in the spleen (Figure 7H). The observation that, during IM-like infection, CD8^+^ T cells appear to control EBV viral loads in blood and spleen, but not in the NALT, suggests that TRMs are less potent than TEMs in controlling primary IM-like EBV infection. Together, these findings suggest a model in which NALT TRMs, while functional, are unable to control robust local B cell proliferation, leading to poor immune control and viral spreading, which is then controlled by more proliferative, CD27-expressing TEMs in blood and spleen.

## Discussion

In this study, we identify a CD8^+^ TRM population that arises in the NALT after i.n. but not i.p. EBV infection of humanized mice. This underscores the importance of initial antigen location for TRM generation in mucosal lymphoid tissues (50, 62). These TRMs accumulate during EBV infection and display phenotypic and functional characteristics similar to TRMs of other tissues. However, in comparison with early differentiated CD27^+^ TEMs that massively expand in lymph nodes and spleen during EBV infection, NALT TRMs express lower levels of CD27 and seem to have limited capacity to restrict EBV infection in submucosal secondary lymphoid tissues. EBV might have adapted to initial infection at submucosal secondary lymphoid tissues that allow for frequent viral reactivations and reinfections (63, 64).

Even prior to infection, the NALT contains a low number of TRMs. This indicates its role as a niche for mucosal TRMs, similar to other lymphoid tissues adjacent to body barriers or long-term niches such as the bone marrow (65–67). Following EBV infection, we detected an increase in the TRM population. This is in line with previous studies demonstrating TRM expansion in the NALT after influenza infection (67, 68). It was additionally shown that EBV-specific T cells can comprise up to 20% of all T cells within the tonsils (36). This T cell accumulation might be driven by EBV-induced B cell proliferation during IM-like primary infection (69, 70). EBV-infected B cells within the NALT seem to expand similarly during infection, which likely leads to enhanced T cell recruitment and stimulation in the NALT.

The NALT is considered an inductive site for humoral and cellular immune responses in the upper respiratory tract (39, 71). Following respiratory virus infections, CD103^+^ T cells are induced in the NALT as well as throughout the nasal turbinate and septum (66). Human studies have shown that CD103^+^ TRMs locate within the tonsillar lymphoepithelial barrier, indicating a patrolling function toward the epithelium (37). The retention of TRMs within the NALT is likely achieved by expression of CD69, CD103, and other integrins including CD11a, which is known to be associated with the retention of memory T cells in other mucosal tissues, such as the lung (72). Compared with previously described TRMs, we could not confirm any specific chemokine receptor expressed by NALT TRMs. They did not express CXCR6, which was previously defined for TRM homing to lung (73). While a subset of TRMs in the NALT expressed *CXCR4* transcripts, the respective protein was not detected by flow cytometry (data not shown). However, in a skin model, it was shown that CXCR4 is dispensable for TRM migration (74). The transcriptional profile of EBV-induced NALT TRMs is consistent with previously described TRM populations, including the expression of TRM core transcription factors *PRDM1* (BLIMP-1), *RUNX3*, and *BHLHE40*. BLIMP-1 and RUNX3 were previously shown to be indispensable for TRM development and function, while BHLHE40 was identified as a master regulator for TRM metabolism (55, 56, 58). Thus, the NALT appears to serve as a niche that allows the development of bona fide TRMs.

In the setting of IM-like disease in humanized mice, NALT-localized T cells did not appear to be essential for local viral control. We recently reported that CD27^+^ T cells are protective against EBV (46) and that CD27 deficiency — as well as loss of its ligand, CD70 — in humans is associated with persisting EBV viremia and associated pathology (75, 76). Recently, CD27 was additionally described to be 1 of 5 markers predictive for EBV-specific T cells within human blood samples (61). Intriguingly, we observed that NALT T cells expressed lower levels of CD27 than their counterparts in the blood and spleen, potentially explaining their reduced EBV-specific immune control in this setting. Similarly, it has been suggested that TRMs are less efficient in the immune control of EBV-infected B cells in the CNS of patients with MS but might contribute to the inflammatory environment in the CNS during this autoimmune disease (77).

Efficient TRM generation and function seem to depend on conditions of intermediate inflammation. Indeed, reduced T-bet expression among effectors has been shown to favor TRM, as opposed to TEM, establishment (78). Since IM represents a more inflammatory manifestation of primary EBV infection, it is possible that this environment favors the establishment of TEM or TEMRA-like T cell memory. Indeed, we observed enhanced TRM generation in a low-dose infection model where viral replication is more moderate and T cell expansion more gradual (45). Similarly, in the context of IM-like EBV infection, T cells express inhibitory molecules including PD-1 and LAG-3, which could be used by EBV-infected B cells to inhibit their function. Interestingly, previous work has demonstrated that, in the tonsils of individuals with IM, fewer memory T cells, including TRMs, are established compared with the tonsils of asymptomatically infected viral carriers (36). These findings are consistent with a scenario where more inflammatory disease prevents efficient establishment of TRMs. It would be interesting to assess whether this further correlates with later indicators of poor EBV immune control such as prolonged viral shedding in the throat, but not blood, of patients with IM (79) or increased incidence of EBV-associated malignancies. Perhaps efficient TRM establishment is important in the later, long-term control of such EBV reactivation-associated conditions.

The fact the EBV-elicited TRMs observed in this study lacked expression of costimulatory molecules crucial for EBV-specific immune control could explain the unchanged viral titers upon T cell or CD103^+^ TRM depletion in the NALT. Notably, our model may not reveal protective TRM functions later during persistent EBV infection; EBV infections in humanized mice were only possible for up to 6 weeks, at which time point viral loads peak in patients with IM and plateau in humanized mice (12, 46, 80). Due to immune pathology in response to this high viral load, EBV-infected humanized mice needed to be euthanized at this time point. This prevented assessment of TRM phenotype and function at later time points when a lower setpoint of persistent viral loads is reached in most patients with IM. However, after 6 weeks of infection with plateauing EBV loads, the NALT nearly uniformly contained T cells with a tissue-resident phenotype. In contrast, in tonsils from presumably healthy virus carriers, a mixture of T cell subsets was observed but was enriched for TRMs compared with spleens of humanized mice after 6 weeks of EBV infection. Notably, this mixture contains CD8^+^ T cells with higher proliferative potential expressing costimulatory molecules of early differentiated CD8^+^ T cells, including CD27, that are required for EBV-specific immune control (46). In contrast, in this context, more terminally differentiated TRMs — predominating in the NALT with reduced proliferative capacities (81) — do not seem to be beneficial in controlling EBV infection and associated pathology, despite similar clonal TCR-Vβ expansions compared with spleens of the same animals. This could explain the diminished immune control of EBV in the mucosa during IM, resulting in prolonged viral shedding into the saliva for months compared with control of viral loads in the blood within weeks after peak viremia (79). Taken together, our results, along with evidence from primary immunodeficiencies and humanized mouse infections that highlight early differentiated and highly proliferative TEMs as being required for EBV specific immune control (46, 76, 82), suggest that further differentiated TRMs with more limited expansion capacity are less protective against EBV infection.

## Methods

### Sex as a biological variable.

For all experiments, animals (described below) were distributed into different experimental groups with a similar ratio of males and females. No differences in titers or expression of key molecules of interest (i.e., CD69, CD103, cytokines or chemokines) were observed by sex.

### Humanized mouse model.

NSG mice and NSG-A2 mice were maintained in ventilated, specific pathogen-free conditions at the Institute of Experimental Immunology, University of Zurich. Newborn pups were reconstituted with human CD34^+^ hematopoietic progenitor cells (HPCs), derived from human fetal liver tissue (HFL) as previously described (45). For each experiment, animals were reconstituted from a single HFL donor and distributed into different experimental groups with similar reconstitution levels of human immune cell populations.

### EBV infection and course of experiment.

Mice were infected with 1 × 10^5^ Raji Green units (RGU) of B95-8 EBV-expressing luciferase under the control of the latent EBNA2 locus (Luc-EBV) (45, 46). Since patients with IM have been found to shed approximately 1 × 10^6^ to 1 × 10^7^ viral DNA equivalents per mL of saliva for prolonged periods after infection, this dose represents the transfer of approximately 10–100 μL of saliva (79). Luc-EBV producer cells were provided by Wolfgang Hammerschmidt (Helmholtz Zentrum, Munich, Germany). Since initial histological examination of the nasal sinuses of uninfected animals revealed heterogeneous establishment of NALT structures (Supplemental Figure 1, A, B, and D), we pretreated animals with SEB (62.5 ng/μL in PBS) applied i.n. to mimic bacterial colonization of mucosal surfaces driving postnatal NALT development in mice (48, 49). A similar approach (i.n. application of *Propionibacterium* *acnes* bacteria) has been used to establish of NALT structures in NALT-deficient CXCR5^–/–^ mice (83). Both lymphocyte recruitment (Supplemental Figure 1, B, C, F, and G) and reproducibility of i.n. infections were significantly improved by SEB pretreatment (Supplemental Figure 1E). EBV infections were performed i.p. or i.n. 2 weeks following SEB pretreatment. For infections, Luc-EBV was applied by 100 μL injection (for i.p. infection) or 20 μL slowly pipetted and divided between both nostrils (for i.n. infection), respectively. Mice infected i.n. were anesthetized with aerosolized isoflurane prior to administration of EBV. Following infection, mice were monitored regularly for 4–6 weeks. Intravascular staining was performed by i.v. injection of 6 μg fluorescent antibody-conjugated CD45 (HI30, BioLegend) diluted in 100 μL PBS 3 to 5 minutes before euthanasia. In each experimental group, 3–6 biological replicates were tested.

### In vivo bioluminescence imaging.

The progression of EBV infection was monitored longitudinally every week and quantitatively measured by in vivo bioluminescence imaging with the IVIS Spectrum Imaging System (PerkinElmer). Animals were anesthetized by aerosolized isoflurane and injected i.p. with 150 mg/kg D-Luciferin (Promega) diluted in PBS 10 minutes before imaging. Mice were placed inside the IVIS imaging box and imaged dorsally and ventrally. Representative images were acquired 10–15 minutes after injection for each mouse throughout each experiment to illustrate viral spread within the host. Images for quantification were captured at various time points before the luminescent signal reached the saturation intensity and analyzed with Living image 4.3.1 software (PerkinElmer). Regions of interest (ROI) were set to include the regions with luminescent signal in mice and photon flux (p/s) of light emitted per second within the ROI was measured as the readout.

### T cell depletion experiments.

CD8^+^ T cells were depleted weekly, via i.p. injection beginning at week 3 after infection, with the monoclonal antibody against human CD8 (clone OKT-8; BioXCell). The initial injection was 75 μg, followed by 2 injections of 50 μg (week 3 after infection) and 3 injections of 50 μg (week 4 after infection). Mice with over 10% residual CD3^+^CD4^–^ T cells within lymphocytes in blood and spleen were considered not depleted and excluded from analysis. CD103^+^ cells were targeted by a single i.p. injection at week 3 with 150 μg anti-CD103 (Ber-ACT8, Ultra-LEAF purified, BioLegend).

### Quantification of EBV DNA genome in blood and tissue.

Total DNA from whole blood was extracted using NucliSENS easyMag (Biomerieux), while solid organs were processed with DNeasy Blood & Tissue Kit (QIAGEN), according to manufacturer’s instructions. TaqMan (Applied Biosystems) real-time PCR was used to quantify EBV DNA as previously described with modified primers for the BamH1 W fragment (5′-CTTCTCAGTCCAGCGCGTTT-3′ and 5′-CAGTGGTCCCCCTCCCTAGA-3′) and a fluorogenic probe (5′-FAM CGTAAGCCAGACAGCAGCCAATTGTCAG-TAMRA-3′). Viral titer concentration was calculated using a standard curve of the international WHO EBV standard, resulting in titers measured in EBV IU. All samples were performed in duplicates and measured on either ViiATM 7 Real-Time PCR System (Thermo Fisher Scientific) or ABI Prism 7300 Sequence Detector (Applied Biosystems). Samples below the lower limit of quantification (LLOQ) of 173 IU/mL were defined as negative for EBV DNA.

Normalization of viral loads was performed to adjust for differences in EBV infection levels between experiments with humanized mice that were reconstituted from different HPC donors.

### Humanized mouse sample collection.

Peripheral blood cells were obtained from animals by tail vein bleeding or terminal heart puncture. Whole blood was lysed twice with 1× ACK lysis buffer for 5 minutes, followed by washing with PBS. Splenocytes were prepared as described above with one time lysis only. NALT, salivary gland, lung, and liver tissues were mechanically disrupted into small pieces and enzymatically digested in 2 mL of digestion buffer (1 mg Collagenase D [Roche] and 0.2 mg DNase I [Roche] in 2 mL RPMI or DMEM) at 37°C for 30 minutes with agitation. Dissociated tissues were then passed through a 70 μm cell strainer. NALT was lysed once with 1× ACK lysis buffer for 3 minutes. Other tissues were partly centrifuged in a discontinuous Ficoll gradient (salivary gland [SG], lung) or Percoll gradient (liver, 40% and 70%; Sigma-Aldrich) for 20 minutes at 1,000*g* using a Sorvall ST 40R centrifuge (Thermo Fisher Scientific). Cells aggregating at the interface between gradient layers were harvested and washed twice with PBS. Bone marrow cells were flushed out of the femur by 5 second pulse centrifugation at 6,000*g*. Cells were washed with PBS and passed through a 70 μm cell strainer if necessary. Cells from blood and spleen were counted using the AcT diff Analyzer (Beckman Coulter) to aliquot the optimal number of cells for staining and calculation of the total cell numbers for different experimental purposes. Calculation of total cell numbers for NALT samples was done using Accucheck counting beads (Thermo Fisher Scientific) as previously described (84).

### Human sample collection.

Human tonsil samples were received from anonymous organ donors in the New York Presbyterian Hospital (New York, New York, USA); 6 tonsils from 4 different individuals were collected immediately after surgery from patients undergoing tonsillectomy for chronic inflammation. Further tonsil samples were received from tonsillectomies performed at the University Hospital of Zurich. Samples were frozen and stored in liquid nitrogen until flow cytometric phenotyping. Samples were rested overnight before stimulation experiments. For comparison of resident versus nonresident populations, tonsil samples were divided into CD69^+^ and CD69^–^ subsamples using biotinylated anti–human CD69 antibody (FN40, BioLegend), and separation was performed using anti-Biotin Microbeads (Miltenyi Biotec, 130-090-485) according to manufacturer’s protocol. Gating during later flow cytometric analyses was chosen according to unstimulated controls from the same samples (Supplemental Figure 6).

### Flow cytometry and antibody and MHC pentamer labeling.

For surface staining, cells were incubated with anti–human FcR-block (Miltenyi Biotec, 130-059-901), live/dead (Zombie NIR/Aqua, BioLegend, 423106 and 423102), and chemokine-receptor antibodies (listed below) for 15 minutes at room temperature. Cells were subsequently incubated with surface marker antibodies for 30 minutes at 4°C. For intracellular and intranuclear labeling, surface staining was followed by fixation and permeabilization with the Foxp3/Transcription Factor Staining Buffer Set (eBioscience, 00-5523-00) according to manufacturer instructions. Antibodies for intracellular targets were incubated for 1 hour at room temperature. All samples were acquired using DIVA software (BD Biosciences) on LSRFortessa/FACSymphony (BD Biosciences) instruments, and analysis was performed using FlowJo software (Tree Star Inc.).

Antibodies used in this study include the following: CD3 (UCHT1, BD Biosciences, BUV661), CD4 (SK3, BD Biosciences, BUV469 / S3.5, Thermo Fisher Scientific, PE-Cy5.5), CD8 (SK1, BioLegend, BV650), CD19 (HIB19, BioLegend, PE-Cy5), CD27 (LG.3A10, BioLegend, BV650), CD39 (A1, BioLegend, BV711), CD45 (HI30, BD Biosciences, BUV395), CD45RA (HI100, BioLegend, APC-Fire750/BV785), CD62L (DREG-56, BioLegend, PE-Cy7/SK11, BD Biosciences, BV510), CD69 (FN50, BioLegend, BV421), CD103 (Ber-ACT8, BioLegend, BV711), CD107a (H4A3, BD Biosciences, FITC), CD127 (IL-7R) (A019D5, BioLegend, Alexa Fluor 700/PE-Dazzle 594), CD279 (PD-1) (EH12.1, BD Biosciences, BUV737), CD335 (NKp46) (9E2, BioLegend, BV510), CD56 (B159, BD Biosciences, APC), Blimp-1 (IC36081R, R&D, Alexa Fluor 647/6D3, BD Biosciences, PE-CF594), CCL5 (VL1, BioLegend, PE), granzyme B (GB11, BioLegend, PE-CF594/Alexa Fluor 700), HLA-DR (G46-6, BD Biosciences, PE-CF594/BV605), IFN-γ (4S.B3, BD Biosciences, BV786), KLRG1 (13F12F2, eBioscience, Alexa Fluor 488), Perforin (dG9, BD Biosciences, PerCP-Cy5.5), TNF-α (Mab11, BD Biosciences, PE-Cy7), anti–mouse TCR-β (H57-597, BioLegend, BV510/605)

EBV-pentamers specific for BMLF1 (GLCTLVAML, HLA-A*02:01, PE/APC) and LMP2 (CLGGLLTMV, HLA-A*02:01, APC/PE) were purchased from Proimmune. Pentamers were added 15 minutes prior to surface antibody labeling during FcR-blocking and live/dead staining.

### Human TCR-Vβ repertoire analysis using flow cytometry.

Human TCR-Vβ repertoire analysis was performed using antibodies against the most frequent TCR-Vβ variants (covering about 70% normal human TCR-Vβ repertoire, as reported by Beckman Coulter Beta Mark TCR vBeta Repertoire Kit; catalog IM3497) and following previously published nomenclature (85). The antibodies were provided by Roland Martin (University of Zurich, Zurich, Switzerland). In brief, frozen splenocytes were stained as described above using surface antibodies against following TCR-Vβ segments. The following were used: VB3 (CH92, Beckman Coulter, FITC), VB4 (WJF24, Beckman Coulter, PE), VB5.2 (36213, Beckman Coulter, FITC), VB5.3 (3D11, Beckman Coulter, PE), VB7.1 (ZOE, Beckman Coulter, PE), VB7.2 (REA677, Miltenyi Biotec, APC), VB9 (FIN9, Beckman Coulter, PE), VB12 (VER2.32.1.1, Beckman Coulter, PE), VB13.1 (IMMU222, Beckman Coulter, PE), VB13.6 (JU74.33, Beckman Coulter, FITC), VB14 (CAS1.1.3, Beckman Coulter, PE), VB16 (TAMAYA1.2, Beckman Coulter, FITC), VB18 (BA62.6, Beckman Coulter, PE), VB20 (ELL1.4, Beckman Coulter, PE), VB21.3 (IG125, Beckman Coulter, FITC), VB22 (IMMU546, Beckman Coulter, FITC), and VB23 (AF23, Beckman Coulter, PE)

After initial analysis, we identified the top 6 TCR-Vβ clones expanded in splenocyte-derived CD8^+^ TEMs in 3 individuals. These were chosen to stain splenocytes and lymphocytes from the NALT of the same mouse for the following TCR-Vβ segments to identify any overlap of clonotypes: VB1 (BL37.2, Beckman Coulter, PE), VB2 (RE654, Miltenyi, PE-Vio770), VB5.1 (LC4, eBioscience, APC), VB8 (56C5.2, Beckman Coulter, FITC), VB11 (RE559, Miltenyi, PE-Vio770), and VB17 (E17.5F3.15.13, Beckman Coulter, FITC).

### Generation of EBV-specific TCR transgenic T cells.

EBV-specific TCR generation and adoptive T cell transfer experiments were performed as previously described (45, 46). Briefly, for each specificity, a total of 200,000 TCR^+^CD3^+^ T cells was transferred i.v. into HPC donor–matched recipient mice and monitored longitudinally during the course of EBV infection.

### scRNA-Seq.

scRNA-Seq of up to 10,000 sorted TEMs was performed using 10X Genomics 3′-kit (v3.1) and Illumina Novaseq S1. Sequencing was performed by the Functional Genomics Center Zurich. Analyses were done with the guidance of NEXUS Zurich and following the OSCA handbook and publication (86). Reads were aligned to a combined reference genome comprising human and EBV reference using the STAR incorporated within the Cell Ranger software (87). Using R 4.0.5, data were quality controlled and normalized to cell cycle genes and within samples before further analysis (88). With corrected values and Poisson residual principal component analysis (PCA), t-distributed stochastic neighbor embedding (t-SNE), and UMAP calculations were done. Subsequent analyses were performed using BioConductor 3.12 unless stated otherwise (86). Unsupervised clustering was performed using the R-implementation of the Phenograph-algorithm (52), and cellular trajectories were calculated with the TSCAN algorithm (53).

### Ex vivo stimulation experiments.

Ex vivo isolated and rested CD8^+^ T cells or transduced T cells from splenocytes were incubated with R10 (RPMI 1640 + 10% FBS + 1% penicillin/streptomycin + L-glutamine + 20 U/mL of IL-2; Thermo Fisher Scientific) alone or R10 containing PMA/ionomycin for 2 hours, followed by the addition of Brefeldin A and Monensin and further incubation for an additional 3 hours. Cells were stained for intracellular cytokines and acquired on a BD FACSymphony. For CD107a labeling, this antibody was added at the very start of coculture. Gating was chosen according to unstimulated controls of the same samples (Supplemental Figure 6).

### Histology.

Whole mouse skulls were fixed using 4% formalin before cutting with a diamond blade. Pieces were decalcified in EDTA at a pH of 9.0 for 20–30 minutes at 100°C and embedded in paraffin. Histological staining was performed by an outside laboratory (Sophistolab AG). In brief, 3 μm sections were processed on a Leica BOND-MAX or Bond-III automated IHC system. Samples were stained with horse radish peroxidase– or alkaline phosphatase–labeled antibodies for 30 minutes at room temperature. The following were used: rabbit α–human CD20 (SP32, Cell Marque), mouse α–human CD20 (L26, Dako), rabbit α-CD3 (SP7, Diagnostic Biosystem), rabbit α–human CD103 (EPR4166[2], Abcam), rabbit α–mouse Lyve-1 (polyclonal [103-PA50AG], RELIATech GmbH), and rat α–mouse/human PNAd (MECA-79, BioLegend). For detection of horse radish peroxidase or alkaline phosphatase, BOND Polymer Refine (DAB) or BOND Polymer Refine Red (Fast Red) were used, respectively (both from Leica Biosystems). EBER in situ hybridization was performed using a Benchmark Ultra automated slide stainer (Ventana). Briefly, tissue sections were pretreated with protease 3 before incubation with an EBER-specific probe (Ventana), which was detected via the ISH iView Blue Detection Kit (Ventana). Images were acquired with the slide imaging PerkinElmer Vectra 3 system. Cell segmentation, total cell count, and phenotype quantification was done using the inform 2.5.1 Tissue Finder Advanced Image Analysis Software from PerkinElmer, as previously described (89). For the final analysis, only cells that the software identified with a confidence level of > 90% were included in the quantification.

### Statistics.

Data were analyzed and graphed using Prism Software (v9.5.1 or 8.3, GraphPad). If not described otherwise, data sets of 2 were compared using 2-way Mann-Whitney *U* test, and data sets of 3 were compared by Kruskal Wallis test with Dunn’s correction for multiple comparisons. Data within same individuals or samples were compared using paired Wilcoxon signed-rank test. For all statistical tests, *P* values less than 0.05 were considered significant. For graphing, either mean ± SD or geometric mean and 95% CI were displayed.

### Study approval.

All animal work strictly followed the animal protocols ZH159/2017 and ZH041/2020, licensed by the Veterinary Office of the Canton of Zurich (Zurich, Switzerland). Human tonsil samples collected at New York Presbyterian and the University of Zurich (Zurich, Switzerland) were obtained as part of IRB-approved protocols within previous investigations (90). All participants provided informed consent in accordance with the Declaration of Helsinki, and the institutional ethics committees (New York Presbyterian and the University of Zurich) approved all protocols used (90).

### Data availability.

Underlying data are provided in the Supporting Data Values file. Raw and processed scRNA-Seq data have been uploaded onto the European database (Annotare/ArrayExpress) and are available under the accession no. E-MTAB-13853 (https://www.ebi.ac.uk/biostudies/arrayexpress/studies/E-MTAB-13853).

## Author contributions

DK, KZ, and CM conceived and designed the experiments. DK, KZ, YD, LR, MB, PS, ADVC, and OC performed the experiments. DK, KZ, MB, and FL analyzed the data. YD, AM, NC, and BC contributed reagents and materials. DK, KZ, and CM wrote the manuscript.

## Supplementary Material

Supplemental data

Supporting data values

## Version 1 09/12/2024

In-Press Preview

## Version 2 10/22/2024

Electronic publication
