# Supplementary material for: Epstein-Barr virus infection induces tissue-resident memory T cells in mucosal lymphoid tissues
Source: JCI Insight. 2024 Oct 22;9(20):e173489. doi: 10.1172/jci.insight.173489 (PMC11530129; doi:10.1172/jci.insight.173489)
Supplement: Supplemental data [file jciinsight-9-173489-s202.pdf]

Epstein Barr virus infection induces tissue-resident memory T cells in mucosal lymphoid tissues

Daniel Kirchmeier[1], Yun Deng[1], Lisa Rieble[1], Michelle Böni[1], Fabienne Läderach[1], Patrick Schuhmachers[1], Alma Delia Valencia-Camargo[1], Anita Murer[1], Nicole Caduff[1], Bithi Chatterjee[1], Obinna Chijioke[2,3], Kyra Zens*[1] and Christian Münz*[1]

[1]Viral Immunobiology, Institute of Experimental Immunology, University of Zürich, Zürich, Switzerland

[2]Cellular Immunotherapy, Institute of Experimental Immunology, University of Zürich, Zürich, Switzerland

[3]Institute of Medical Genetics and Pathology, University Hospital Basel, Basel, Switzerland

*Equal contribution

Supplementary Figures

Supplementary Figure 1: NALT contains Lyve-1^{+} and PNAd^{+} lymphatic vessels and B and T lymphocytes.

Supplementary Figure 2: EBV can be detected in NALT and submandibular tissue following IN infection.

Supplementary Figure 3: IN EBV infection induces virus-specific TRMs in the NALT.

Supplementary Figure 4: Similar TRM marker expression in both NALT clusters identified by single cell RNA sequencing analysis.

Supplementary Figure 5: Similarities in TRM marker expression between CD8^{+} T cells from EBV-infected NALT and tonsils from EBV-positive donors.

Supplementary Figure 6: Function of TEM derived from NALT during IN EBV infection or from tonsils of EBV carriers.

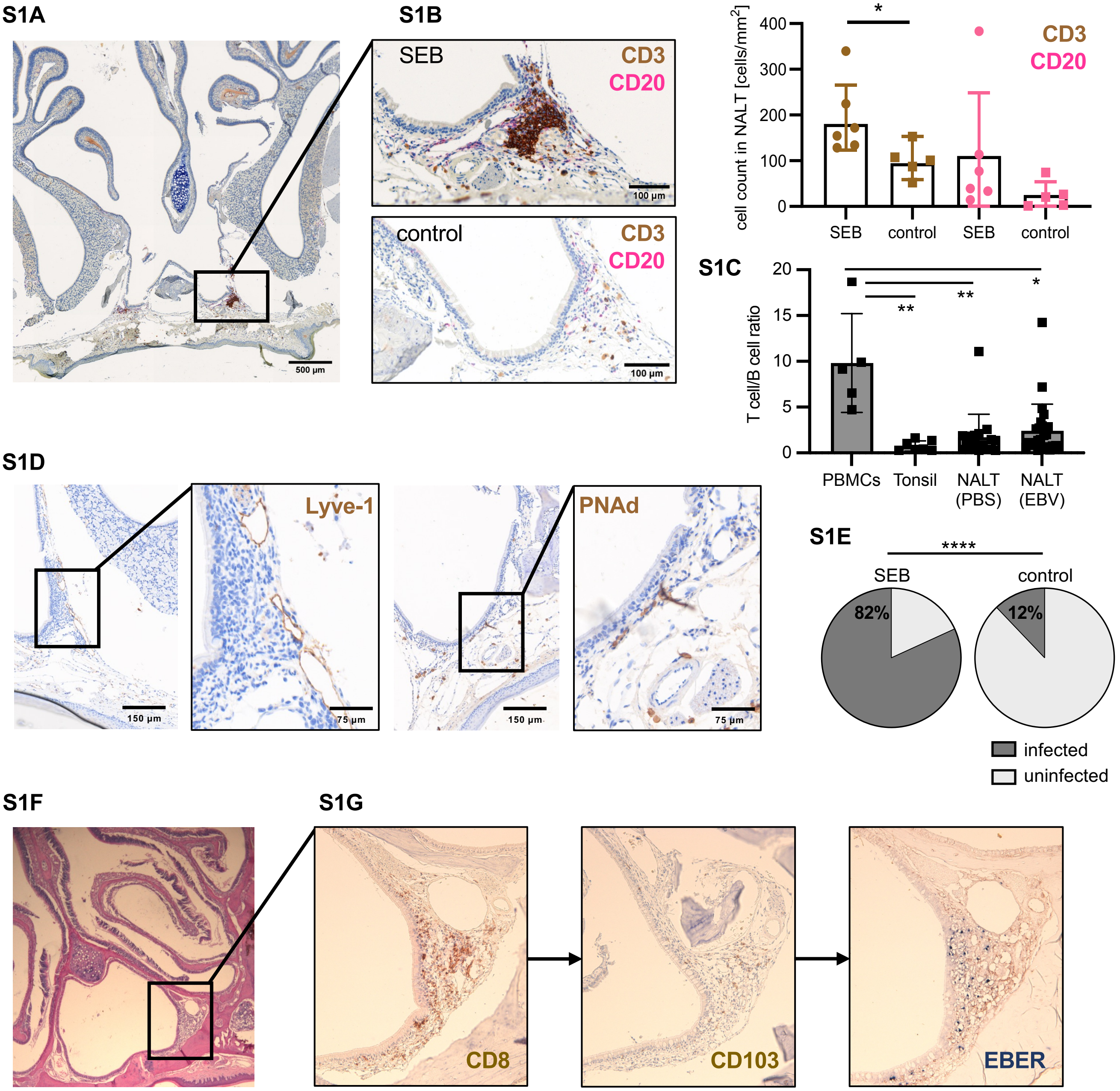

Supplementary Figure 1: NALT contains Lyve-1⁺ and PNAd⁺ lymphatic vessels and B and T lymphocytes. (A) Representative paraffin-embedded PFA-fixed NALT sections stained for CD3 (3,3′-Diaminobenzidine/DAB, brown) and CD20 (Fast Red) for (B) SEB-pre-treated (top) and (bottom) control mice, with (right) quantification of $CD3^+$ and $CD20^+$ cells in the NALT area of SEB-pre-treated and control mice. (C) $CD3^+$ to $CD20^+$ ratios in PBMCs, human tonsils, uninfected but SEB-treated NALT and EBV infected SEB-treated NALT (PBMCs n=5, tonsil n=7, uninfected NALT (PBS mock) n= 17, EBV-infected NALT n=26). (D) Paraffin-embedded PFA-fixed NALT sections stained for lymphatic vessel endothelial hyaluronan receptor 1 (Lyve-1; DAB) (left panels) or (right panels) peripheral node addressin (PNAd; DAB). (E) Quantification of successfully intranasally-infected (dark gray) and uninfected (light gray) individual mice following SEB pre-treatment (infection status = positive titers in blood at end of experiment; n=33-77 animals per group from 2-8 independent experiments). (F) Paraffin-embedded PFA-fixed NALT sections stained for hematoxylin and eosin (H&E) for SEB-pre-treated intranasally EBV-infected mice and (G) further serial sections of the same NALT stained for CD8 (left), CD103 (middle), or Epstein-Barr virus-encoded small RNA (EBER) in situ hybridization (right). (*, $P \leq 0.05$, **, $P \leq 0.01$, ****, $P \leq 0.0001$; Mann-Whitney-U test for CD3 and CD20 comparisons, Kruskal-Wallis ANOVA with Dunn's test for multiple comparisons for comparison of PBMCs, tonsil and NALT, Fisher's exact test for comparison of infection frequencies)

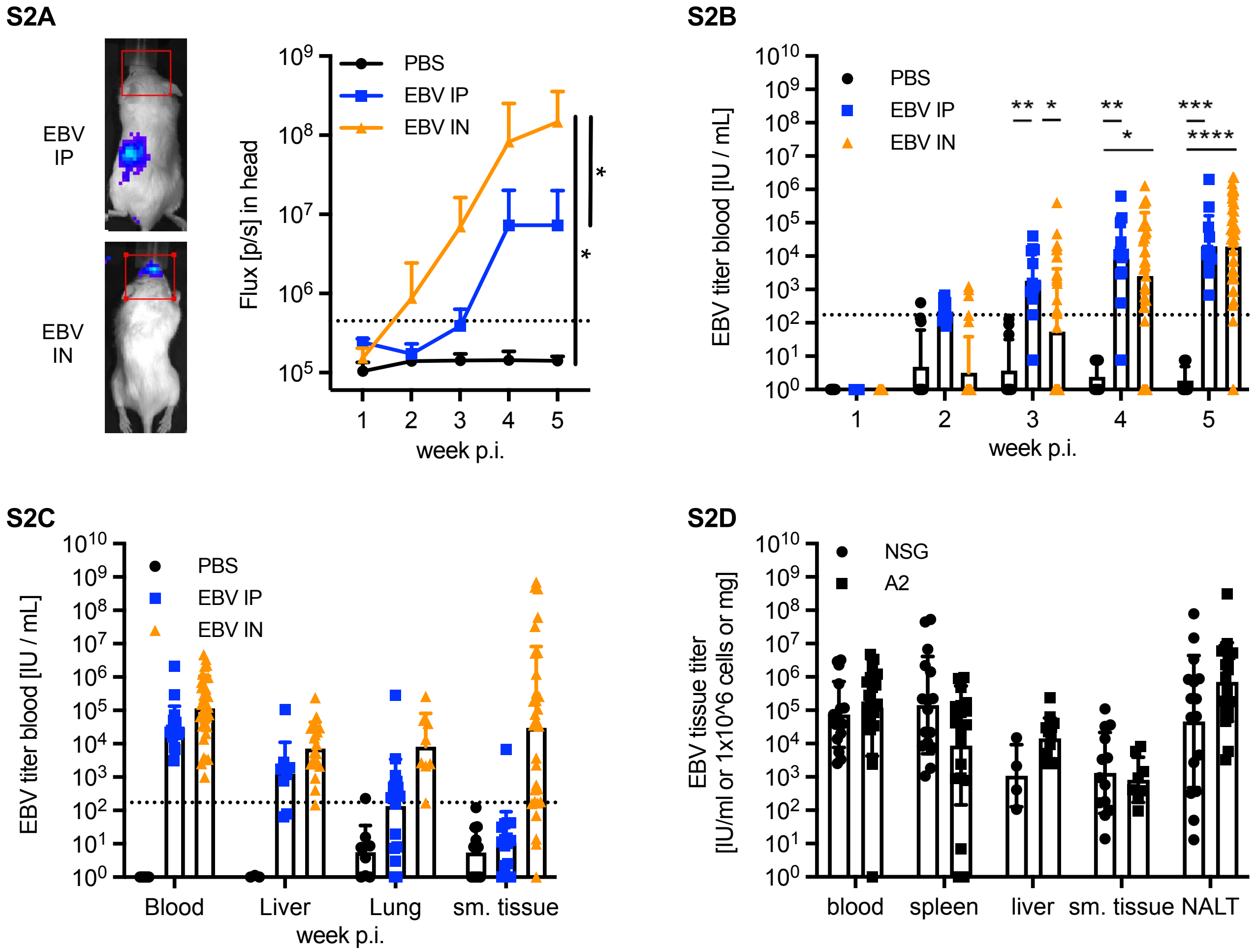

Supplementary Figure 2: EBV can be detected in NALT and submandibular tissue following IN infection. (A) Representative IVIS images 1 week following Luc-EBV infection (left) with (right) quantification of the outlined region of interest (n=9-14 animals per group from 2-3 independent experiments, EBV IP = intraperitoneal infection, EBV IN = intranasal infection, dotted line = background). (B) EBV blood viral loads over time in International Units (IU)/ml in infected animals or PBS controls (n=4-31 animals per group from 3-6 independent experiments; quantification of 1B). (C) EBV viral loads in blood and indicated tissues at sacrifice (n=3-32 animals per group from 3-6 independent experiments; quantification of 1C). (D) EBV viral loads in blood and indicated tissues at sacrifice comparing NSG and NSG-A2 animals (n=4-21 animals per group from 4 independent experiments). (*, $P \leq 0.05$, **, $P \leq 0.01$, ***, $P \leq 0.001$, ****, $P \leq 0.0001$; Kruskal-Wallis ANOVA with Dunn's test for multiple comparisons)

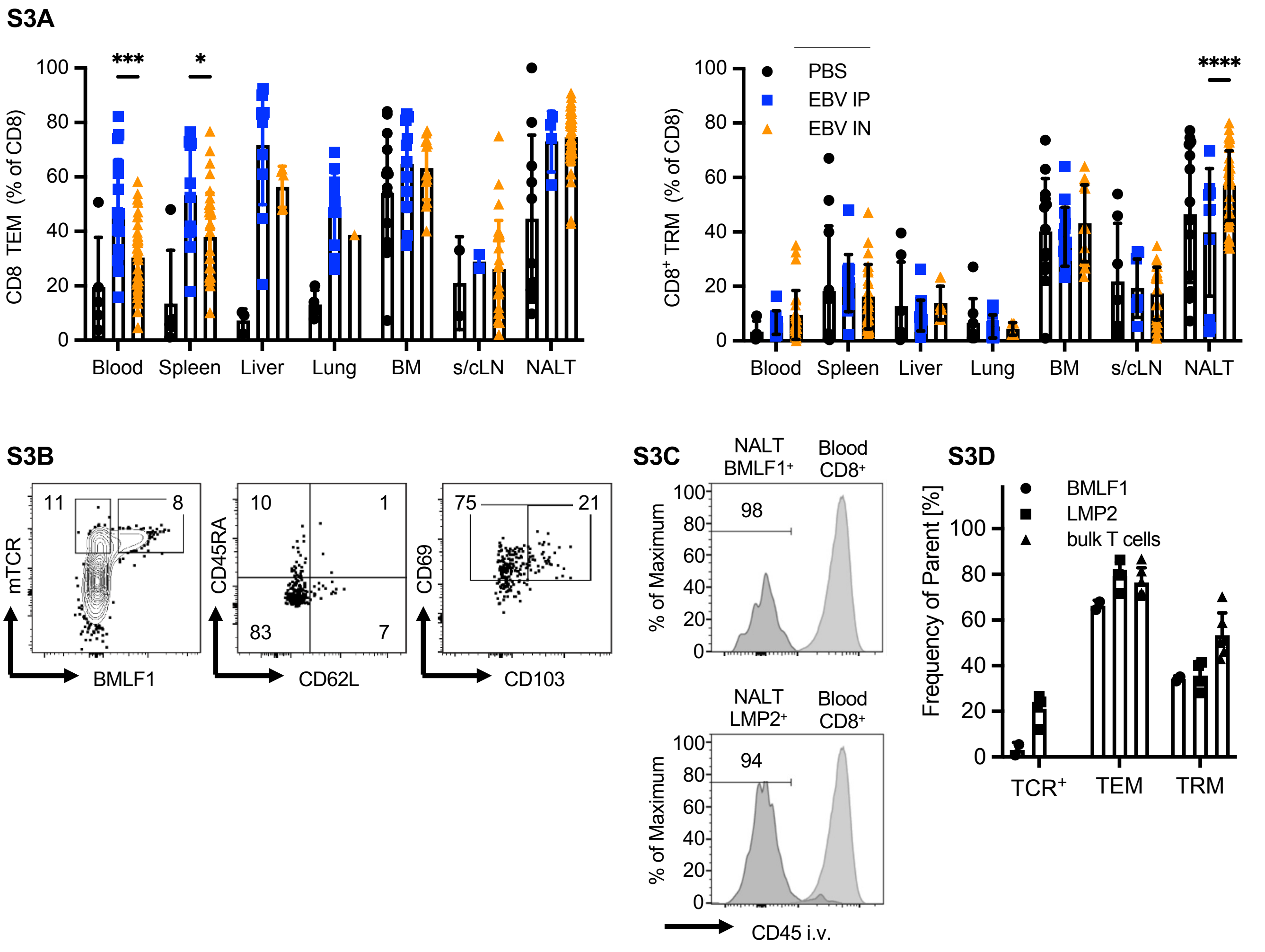

Supplementary Figure 3: IN EBV infection induces virus-specific TRMs in the NALT. (A) Frequencies of CD8+ TEM (left) and (right) TRM (CD69+ TEM) T cells in indicated tissues at sacrifice following i.n. EBV infection (n=4-34 animals per group from a minimum of 2 independent experiments; quantification of 2E). (B) Representative histogram of i.v. injected anti-CD45 on CD8+ T cells from the blood and EBV BMFL1 (top) and (bottom) LMPs-specific T cells in the NALT. (C) Representative flow cytometry plot identifying BMLF1-specific T cells harboring hybrid human/murine transgenic TCR (mTCR) with HLA-A*02:01 plus LMP2 peptide pentamer (LMP2) in the NALT (left) and representative flow cytometry plots of EBV-specific T cells showing (middle) TEM and (right) TRM phenotypes (D) Quantification of EBV-specific TCR-T cell populations in the NALT (n=2-3 animals per group). (*, P ≤ 0.05, ***, P ≤ 0.001, ***, P ≤ 0.0001, Kruskal-Wallis ANOVA with Dunn's test for multiple comparisons)

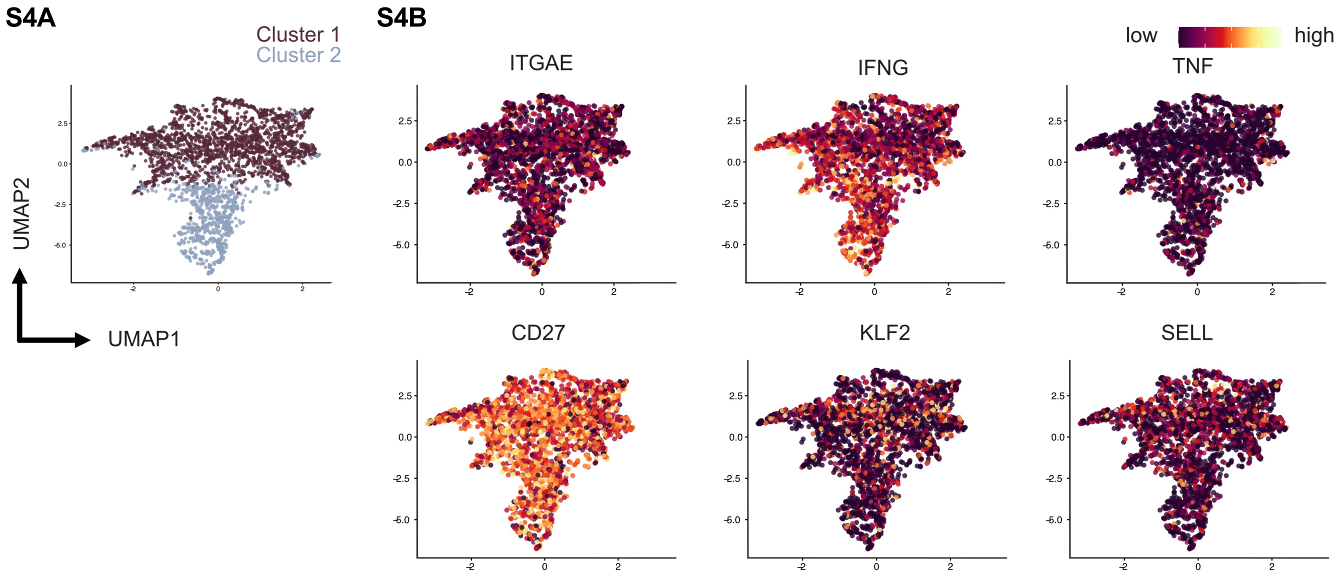

Supplementary Figure 4: Similar TRM marker expression in both NALT clusters identified by single cell RNA sequencing analysis. (A) UMAP plot of single $CD8^+$ TEM transcriptomes sorted from NALT depicting the formation of two distinct clusters. (B) Integrated UMAP plots of cells showing relative expression of selected TRM markers (yellow-green = high expression, violet = low expression).

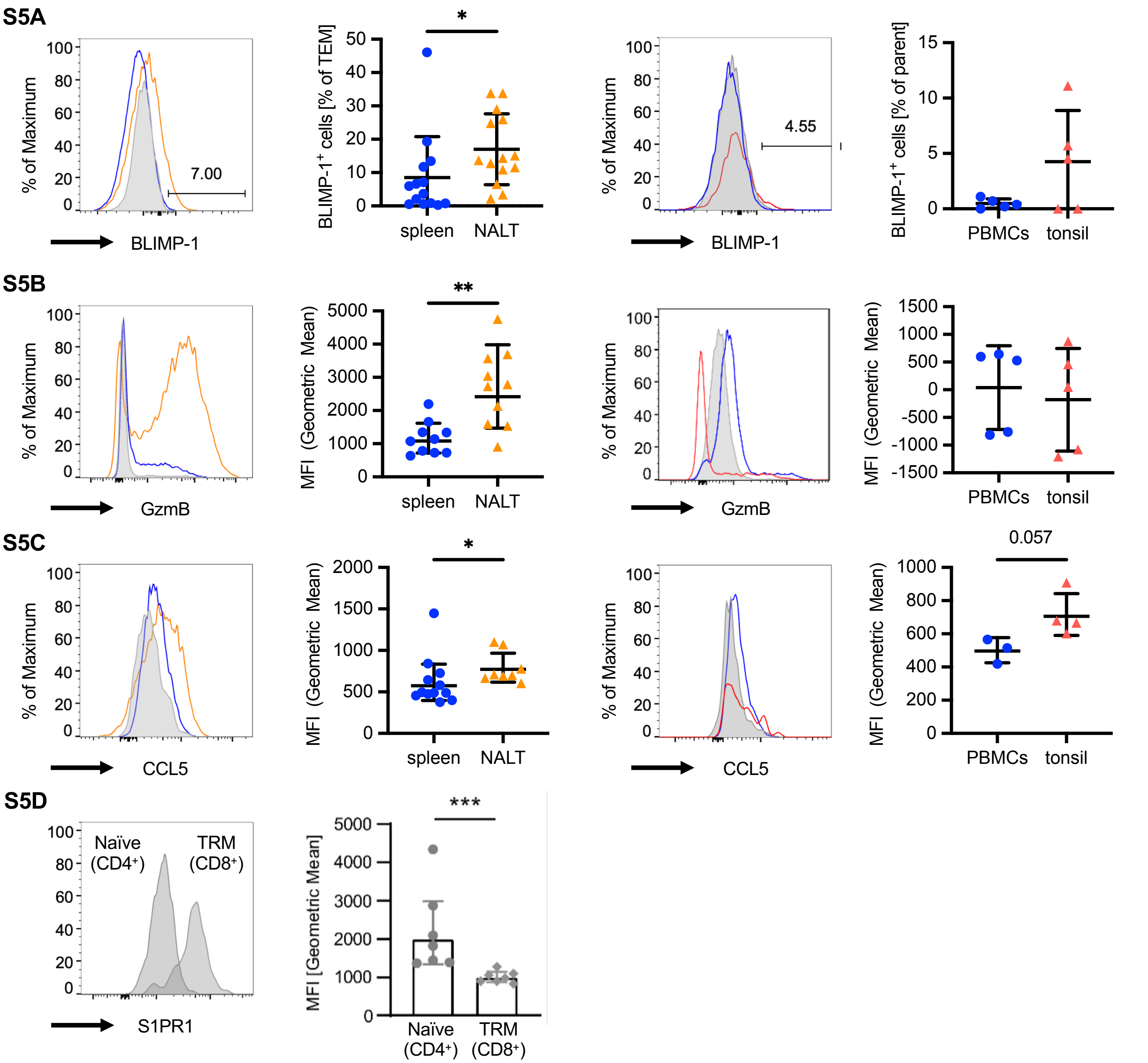

Supplementary Figure 5: Similarities in TRM marker expression between CD8⁺ T cells from EBV-infected NALT and tonsils from EBV-positive donors. (A) Representative histogram of geometric mean fluorescence intensity (MFI) and quantification of BLIMP-1 expression by TEM from spleen (blue) and NALT (orange) or naïve CD8⁺ T cells from spleen (gray) of EBV-infected animals (left two panels) and (right two panels) in TEM from human PBMCs (blue) and tonsils from EBV-positive donors (red) or naïve CD8⁺ T cells from blood (gray). (B) MFI and quantification of Granzyme B (GzmB) expression by TEM in spleen (blue) and NALT (orange) or naïve CD8⁺ T cells from spleen (gray) of EBV-infected animals (left two panels) and (right two panels) in TEM from human PBMCs (blue) and tonsils from EBV-positive donors (red) or naïve CD8⁺ T cells from blood (gray). (C) MFI and quantification of CCL5 expression by TEM in spleen (blue) and NALT (orange) or naïve CD8⁺ T cells from spleen (gray) of EBV-infected animals (left two panels) and (right two panels) in TEM from human PBMCs (blue) and tonsils from EBV-positive donors (red) or naïve CD8⁺ T cells from blood (gray). (D) MFI and quantification of S1PR1 expression in CD8⁺ TRM and naïve CD4⁺ T cells (as a control) derived from NALT of EBV-infected animals. (For animal data, n=7-12 animals per group from at least 2 independent experiments; for human data, n=3-5 individuals per tissue from 2 independent experiments, except CCL5 data which is derived from a single experiment; *, $P \leq 0.05$, **, $P \leq 0.01$; Wilcoxon matched-pairs signed rank test for animal data, Mann-Whitney-U test for human data)

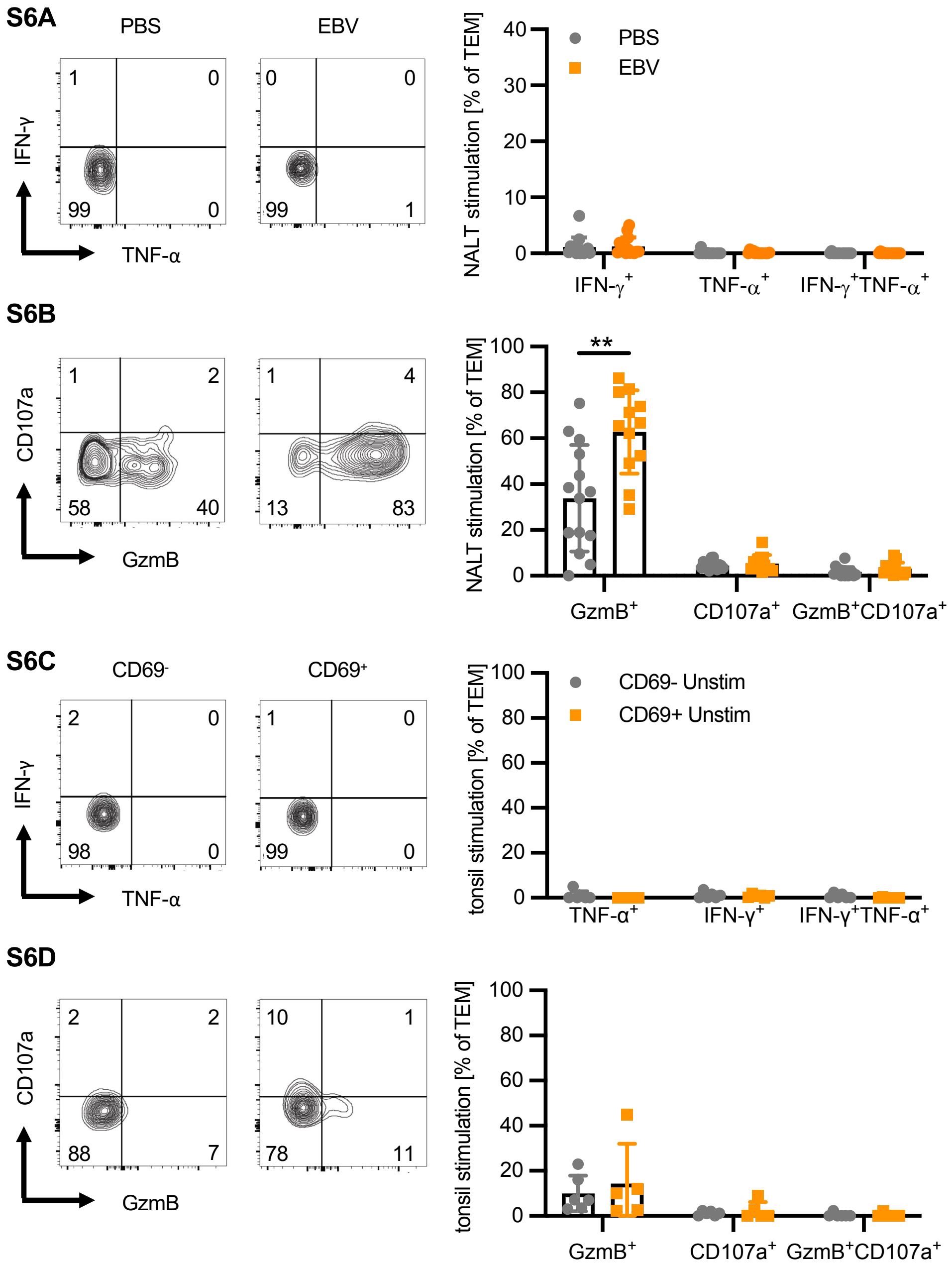

Supplementary Figure 6: Function of TEM derived from NALT during IN EBV infection or from tonsils of EBV carriers. (A) Representative flow cytometry plots showing IFN-γ and TNF-α expression by $CD8^+$ TEMs derived from PBS or EBV infected NALT without stimulation (left panels) and quantification (right panels) of each population. (B) As for (A), but depicting CD107a and Granzyme B (GzmB) expression. (C) Representative flow cytometry plots showing IFN-γ and TNF-α expression by$CD69^-$ or $CD69^+$ $CD8^+$ TEMs derived from tonsils of EBV carriers without stimulation (left panels) and quantification (right panels) of each population. (D) As for (C), but depicting CD107a and Granzyme B (GzmB) expression. (Corresponding stimulated data for all samples can be found in Figure 6, for animal data, n=12-14 animals per group from 2 independent experiments; for human data, n=3-5 individuals per tissue from 2 independent experiments; **, $P \leq 0.01$; Wilcoxon matched-pairs signed rank test for animal data, Mann-Whitney-U test for human data)
